# Synergistic ultraviolet and visible light photo-activation enables intensified low-temperature methanol synthesis over copper/zinc oxide/alumina

**DOI:** 10.1038/s41467-020-15445-z

**Published:** 2020-03-31

**Authors:** Bingqiao Xie, Roong Jien Wong, Tze Hao Tan, Michael Higham, Emma K. Gibson, Donato Decarolis, June Callison, Kondo-Francois Aguey-Zinsou, Michael Bowker, C. Richard A. Catlow, Jason Scott, Rose Amal

**Affiliations:** 1 School of Chemical Engineering, UNSW Australia, Sydney, NSW 2052 Australia; 2 Applied Chemistry and Environmental Science, School of Science, RMIT University, Melbourne, VIC 3000 Australia; 3 UK Catalysis Hub, Research Complex at Harwell, Rutherford Appleton Laboratory, Harwell, Oxon, OX11 0FA UK; 4 School of Chemistry, Cardiff University, Park Place, Cardiff, CF10 1AT UK; 5 School of Chemistry, Joseph Black Building, University of Glasgow, Glasgow, G12 8QQ UK; 6 Department of Chemistry, University College London, 20 Gordon St, London, WC1 HOAJ UK

**Keywords:** Catalytic mechanisms, Heterogeneous catalysis, Photocatalysis, Electron transfer

## Abstract

Although photoexcitation has been employed to unlock the low-temperature equilibrium regimes of thermal catalysis, mechanism underlining potential interplay between electron excitations and surface chemical processes remains elusive. Here, we report an associative zinc oxide band-gap excitation and copper plasmonic excitation that can cooperatively promote methanol-production at the copper-zinc oxide interfacial perimeter of copper/zinc oxide/alumina (CZA) catalyst. Conversely, selective excitation of individual components only leads to the promotion of carbon monoxide production. Accompanied by the variation in surface copper oxidation state and local electronic structure of zinc, electrons originating from the zinc oxide excitation and copper plasmonic excitation serve to activate surface adsorbates, catalysing key elementary processes (namely formate conversion and hydrogen molecule activation), thus providing one explanation for the observed photothermal activity. These observations give valuable insights into the key elementary processes occurring on the surface of the CZA catalyst under light-heat dual activation.

## Abstract (web summary)

CO_2_ to methanol synthesis is a promising approach for renewable fuel production. Here, the authors show that UV and visible light dual activation promotes photothermal methanol production at the copper-zinc oxide interfacial perimeter by accelerating formate conversion and hydrogen molecule activation.

## Introduction

Referred to as a “methanol economy”^1^, methanol (MeOH) synthesis from the chemical recycling of CO_2_ is considered a promising approach to reduce atmospheric CO_2_ levels and the dependence on fossil fuels since MeOH can be readily utilised as a liquid fuel and is well-established as a carbon feedstock material. Despite its potential, the CO_2_-to-MeOH reaction is inherently restricted by thermodynamic limitations and energy-intensive operational conditions^2,3^. While elevated temperatures (>240 °C) favour CO_2_ activation, they are detrimental towards MeOH production due to the exothermic nature of the reaction and the presence of the competitive reverse water gas shift (RWGS) reaction at higher temperatures^4,5^.

Despite the emergence of new catalysts for the CO_2_-to-MeOH reaction^6,7^, the industrially relevant and well-established Cu/ZnO/Al_2_O_3_ (CZA) catalyst (Cu loading of 30–70 at.%) remains one of the most active MeOH-production catalysts in terms of both turn-over frequency (TOF, based on Cu active sites) and MeOH yield. The advantages of the CZA originate from the distinctive ZnO promotion in the stabilisation of intermediates involved in CO_2_ hydrogenation over the Cu–ZnO system^8^. Recent studies on Cu–ZnO catalyst suggested that the Cu–ZnO interface may provide active sites for multiple species or intermediary reactions. Moreover, a Cu–ZnO strong metal-support interaction (SMSI)^9,10^ was identified as playing a key role in the creation and maintenance of surface Cu(I) active sites^11^, with the stabilisation of oxygenates near Cu(I) sites being linked to high MeOH synthesis activity^12^. Lunkenbein et al.^10^ proposed the formation of a chemically bonded Cu^δ+^–O–ZnO interface as the active site for MeOH synthesis based on their structural/componential observations. To date, the kinetically-relevant formate hydrogenation step still represents one of the major bottlenecks in MeOH synthesis via the formate-pathway over ZnO/Cu catalysts. One promising alternative to intensify the HCOO*-conversion is by resorting to a photo-assisted approach.

Plasmon-induced hot electrons are reported to be capable of activating chemical bonds and driving chemical conversion in a well-designed, energetically-matched surface-adsorbate array^13–17^. For example, Zhang et al.^18^ reported that during CO_2_ hydrogenation on a Rh metal surface under irradiation, increased methanation was observed, with CH_4_ being preferred over the kinetic RWGS product, CO. This effect was attributed to electrons selectively being transferred to the antibonding orbitals of HCO species (weakening of the C=O bond). Plasmon-induced H_2_ dissociation on Au^16^, Ni^17^, and Al^19^ metals has also been reported and could be effectively employed in photochemical hydrogenation reactions^20^. However, beyond plasmonic enhancement, the potential exists for non-plasmonic components and even further synergism amongst different photo-activation modes; this is an attractive possibility, particularly in complex catalytic systems (where multiple catalyst components and products are involved). For instance, Zhang et al.^21^ recently published findings that demonstrated a synergy between charge separation on an oxide, and localized surface plasmon resonance (LSPR) induced H_2_ activation on a metal for CO_2_-to-CO conversion under UV-Vis irradiation.

Recently, Wu et al.^22^ found that light irradiation (UV+visible) can be applied to MeOH synthesis over Pd/ZnO catalysts, with an improvement in product yield by 1.5–3 times (up to 3.5 mmol g_cat._^−1^ h^−1^), while operating at a lower pressure. Wang et al.^23^ demonstrated that light-assisted MeOH synthesis (0.06 mmol g^−1^ h^−1^) can be achieved over defect-rich In_2_O_3-x_(OH)_y_ at atmospheric pressure. The semiconducting (ZnO, Cu_2_O, CuO) and potentially plasmonic (Cu) nature of the industrial CZA catalyst components suggest that catalytic performance could be improved by simultaneous thermal-light activation. In addition, the excitations associated with specific components of CZA, namely the ZnO band gap excitation (3.2 eV^[ 22^ = ~388 nm), and Cu LSPR (~590 nm^24^), will change the electron-populating state of catalyst, thus regulating the intermediate reactions and product distribution. The effects of band gap excitations associated with Cu_2_O and CuO components (2.2 eV and 1.7 eV, respectively^25^) are negligible due to the very limited presence of these components after reduction^26^.

In this work, we investigate the effect of electronic excitations on photothermal catalytic CO_2_ hydrogenation over a Cu/ZnO/Al_2_O_3_ (CZA) catalyst by probing the surface to explore light-triggered surface reactions via X-ray spectroscopies and in situ diffuse reflectance infrared Fourier transform spectroscopy (DRIFTS). The key elementary catalytic processes (CO_2_ deoxygenation, H_2_ dissociation and migration, HCOO* conversion, etc.) are examined to provide explanation in the variation of catalytic performance and surface chemistry. In addition, the relative role of Cu and ZnO sites in CZA-catalysed MeOH synthesis is also scrutinised.

## Results

### Light-assisted CO_2_ hydrogenation performance

**Fig. 1:**
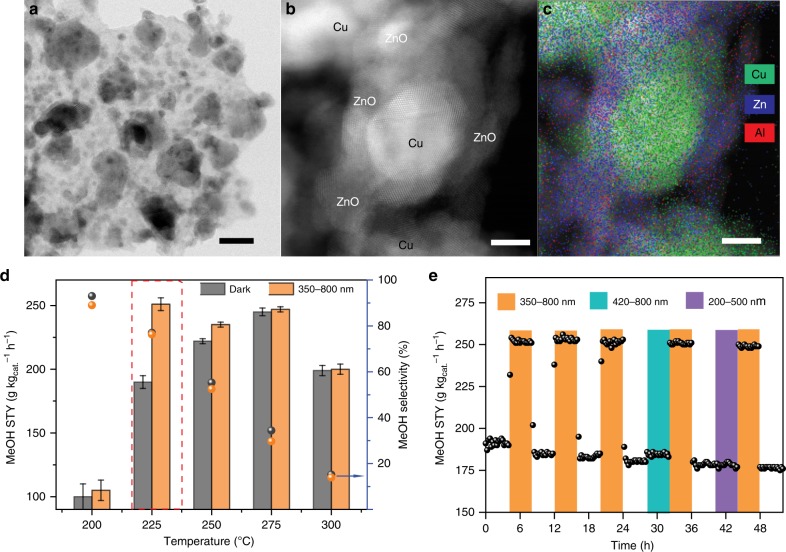
Structural properties and catalytic activities. **a**, **b** HR-TEM images and **c** EDS map of reduced CZA catalyst, scale bar, 25 nm (**a**) and 5 nm (**b**, **c**); **d** MeOH selectivity/space time yield (STY) at 200–300 °C under dark and 350–800 nm light irradiation (red dotted box indicates the highest photo-enhancement at 225 °C). Error bars indicate the deviation among three independent experiments; **e** time-on-stream MeOH yield under different light irradiation conditions (350–800 nm, 200–500 nm, and 420–800 nm, which corresponds to the excitation of Cu, ZnO, and Cu + ZnO, respectively). Reaction conditions: CO_2_: H_2_ = 1: 3.2, GHSV = 8758 h^−1^, P = 21 bar, T = 225 °C, light intensity = 600 mW cm^−2^ for all spectral ranges.

The CZA catalyst comprises spherical copper nanoparticles (10 ± 3 nm) surrounded by smaller ZnO nanoparticles (Fig. 1a–c and Supplementary Fig. 2), consistent with the previous reports^7,27^. Further details on the structural properties of CZA-related samples are provided in the Supplementary Information (Supplementary Note 1, Supplementary Figs. 1–5 and Supplementary Table 1). Temperature programmed CO_2_ hydrogenation was conducted in a Harrick reactor at 21 bar under both dark and irradiated conditions. Catalytic performance was assessed by the MeOH space time yield (STY) and MeOH selectivity (Eqs. 1 and 2 in the “Methods” section).

Without light irradiation (thermal catalytic reaction), the light-off curve for MeOH STY exhibited a volcano-like profile, which peaked at 275 °C (245 g kg_cat._^−1^ h^−1^). The volcano-like profile is attributed to a steady decrease in MeOH selectivity from 93% to 15.3% as temperature is ramped from 200 °C to 300 °C. When irradiated with 350–800 nm light, significant photo-enhancement in MeOH production was only observed at 225 °C (32%), corresponding to the highest MeOH STY with only a slight change in product selectivity. In addition, compared to the performance at 275 °C in the dark, 350–800 nm irradiation enables a 50 °C decrease in reaction temperature for the same MeOH yield while simultaneously delivering a much higher selectivity (>75%) toward MeOH (Fig. 1d, see also Supplementary Note 2). More importantly, the improved MeOH selectivity suggests that the photo-enhancement is more than a simple heating effect from light irradiation (see also Supplementary Fig. 6 and Supplementary Note 3).

To understand the role of the individual photo-responsive components in the CZA catalyst, isothermal CO_2_ hydrogenation performance by CZA was assessed at 225 °C. Three different band ranges were employed: 200–500 nm, 420–800 nm, and 350–800 nm. The 200–500 nm spectral range encompassed ZnO (3.2 eV) band gap excitation while the 420–800 nm spectral range covered Cu/CuO_x_ (LSPR and band gap excitation, respectively) (Supplementary Fig. 7). Despite the promotion of CO production under all three irradiation conditions (Supplementary Fig. 8a), MeOH STY was only greatly improved with the irradiation of 350–800 nm range (Fig. 1e). The findings highlight that simultaneous excitation of both Cu and ZnO is crucial to promote synergistic MeOH production, since of the three band ranges employed, only the 350–800 nm spectral range encompasses the key excitations of both components. In addition, the cycling result (Fig. 1e) shows that photo-enhanced MeOH production under 350–800 nm irradiation can be retained with good durability over 48 h when light is reintroduced, regardless of the prior lighting conditions.

### Surface intermediates and the role of the Cu-ZnO interface

**Fig. 2:**
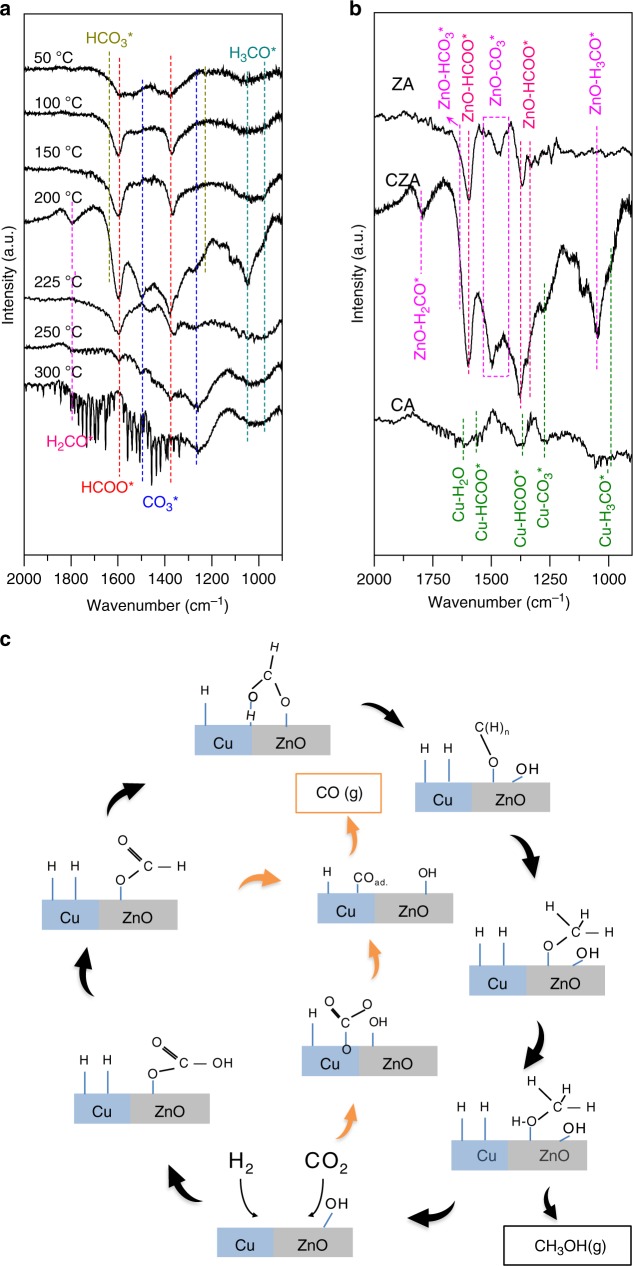
Surface intermediates and reaction diagram. **a** Saturated DRIFTS spectra of different samples at 200 °C; **b** N_2_-purged DRIFTS spectra of CZA recorded at different temperatures (when stabilized at each temperature the catalyst was purged under N_2_ for about 1.5 h to remove MeOH and H_2_O molecules), the formaldehyde (H_2_CO*), formate (HCOO*), carbonate (CO_3_*), bicarbonate (HCO_3_*), and methoxy (H_3_CO*) species are labelled with dotted lines; **c** Proposed methanol (black arrow) and CO production (orange arrow) pathways over Cu-ZnO catalyst.

To elucidate the role of Cu and ZnO in the light-assisted hydrogenation of CO_2_, surface adsorbed intermediates on CZA, Zn/Al_2_O_3_ (ZA), and Cu/Al_2_O_3_ (CA) were probed using in situ DRIFTS (CO_2_ + H_2_, 50–300 °C under 15 bar, Fig. 2a, b, and Supplementary Fig. 9). Examination of the DRIFTS spectra of CZA revealed several adsorbed surface species: (i) carbonate (CO_3_*, 1463–1492 cm^−1^), (ii) bicarbonate (HCO_3_*, 1622 cm^−1^), (iii) formate (HCOO*, 1550 cm^−1^ and 1590 cm^−1^), (iv) methoxy (ZnO-H_3_CO* at 1050 cm^−1^ or Cu-H_3_CO* at 979 cm^−1^), and, (iv) formaldehyde (H_2_CO*, 1795 cm^−1^)^28^. Detailed vibrational peak assignments are available in Supplementary Table 2. Notably, different distributions of surface intermediate species were observed on the surface of ZA and CA, supporting our postulation that the two active sites serve different purposes in the synergistic conversion of CO_2_ to MeOH (see also Supplementary Note 4).

Bowker and Waugh^29^ emphasised the importance of the HCOO* intermediate in Cu driven CO_2_ conversion to MeOH. Hence, to distinguish between HCOO* species adsorbed at different surface sites, herein DRIFTS was performed on catalysts with pre-adsorbed formate species (sodium formate). The DRIFTS spectra (Supplementary Fig. 10) indicate that the HCOO* vibrational peak at ~1550 cm^−1^ and ~1590 cm^−1^ corresponds to HCOO* on Cu (Cu–HCOO*) and Zn (ZnO–HCOO*) respectively. The sole vibrational peak of ZnO–HCOO* on CZA suggests that ZnO is the primary active site for CO_2_ conversion to formate, and consequently also plays a role in the activation of formate towards subsequent elementary reaction processes. In addition, we observed a similar vibrational feature on ZA in the range of 1300–1650 cm^−1^, confirming the preferential initial adsorption of CO_2_ on the surface of ZnO^30,31^. The accumulation of Zn–HCOO* on CZA at temperature <250 °C suggest that hydrogenation of the formate species represents one of the most significant kinetic barriers in MeOH-synthesis reactions (and is correlated to the observed temperature-dependent photo-enhancement effect described in Supplementary Fig. 11 and Supplementary Note 5). Nonetheless, conversion of ZnO–HCOO* to MeOH is not favourable in the absence of Cu as suggested by the absence of H_3_CO* species on the surface of ZA (Fig. 2a).

To understand the role of Cu, we compared the DRIFTS spectra of CZA and CA (Fig. 2a). CA exhibits significantly weaker vibrational spectra, predominantly of Cu–OH* (1602 cm^−1^), Cu–HCOO* and Cu–CO_3_*. Weigel et al.^32^ studied CO_2_ hydrogenation on Cu/ZrO_2_ catalyst and showed that CO formation may proceed via CO_3_* dissociation (CO_3_* + H_2_ → CO_gas_ + 2OH*). This observation is consistent with the surface adsorbed species detected on CA. We consider that the CO was produced mainly through CuO_x_–CO_3_* dissociation at lower temperature and was desorbed from the Cu surface^33^ as indicated by the observation of adsorbed CO* species (2077 cm^−1^ and 2094 cm^−1^ on the Cu(111) surface^34^) on CZA and CA catalysts (but not on ZA) (Supplementary Fig. 9). Thus, the CA sample exhibits higher selectivity towards CO compared to that of CZA (Supplementary Fig. 8b). Weak H_3_CO* vibrational peaks can be observed on CA (979 cm^−1^), suggesting that hydrogenation of HCOO* to H_3_CO* is primarily driven by Cu. However, ZnO incorporation intensified this process. Additionally, an accelerated decrease in accumulated HCOO* on CZA compared to ZA (Supplementary Fig. 12) can be observed at T > 225 °C. These phenomena can be attributed to conversion of the ZnO–HCOO* to H_3_CO*, presumably at the interfacial perimeter of Cu–ZnO. Larmier et al.^35^ observed a similar interaction between Cu metal and ZrO_2_ support during CO_2_ conversion to MeOH.

### Computational study

It has been proven that for electron transfer to occur between catalyst and adsorbates, orbital overlap between the substrate and adsorbate is essential^16,36,37^. To explore this possibility, first-principles density functional theory (DFT) calculations were performed to obtain the relative orbital energies for each of the identified reactants, products, and intermediates as free gas phase species. These were then compared to the experimental valence band spectra. Despite its limitations (see also Supplementary Note 6), the current method is unbiased and does not make any particular assumption in terms of the surface structure and composition of the catalyst.

**Fig. 3:**
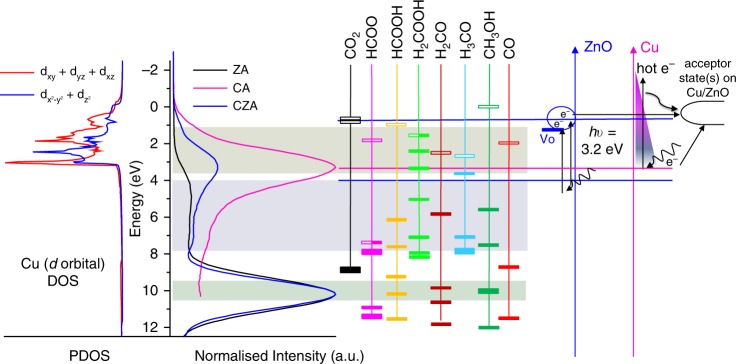
Alignment of gas phase adsorbate orbital levels. In order to compare the calculated orbital energies with the experimental spectra, the calculated orbital energies were aligned such that all share a common vacuum level. The Fermi levels of Cu 3*d* DOS and all reaction intermediates are aligned to the CZA Fermi level using a catalyst work function value of 4.7 eV, typical of ZnO and Al_2_O_3_. The aligned orbital levels and the experimental valence band spectra are depicted, hence *E* = 0 eV corresponds to the CZA Fermi level with the common vacuum level at −4.7 eV (not shown). The electron-excitation under irradiation and its transfer to surface acceptor states in ZnO and schematic depiction of LSPR-induced indirect/direct hot electron transfer from Cu to adsorbate.

The vacant CO_2_ orbital levels at 0.79 eV and 0.45 eV correspond to the C–O σ* antibonding orbital and two degenerate C–O π* antibonding orbitals, respectively. Filling of these orbitals by electron transfer from the substrate is a prerequisite for any degree of chemisorption, and activation towards subsequent reactive processes (i.e. hydrogenation or dissociation). Whilst these levels are lying higher than the main Cu 3*d* valence band, they correspond well with the ZnO excited state (i.e. the dominant ZnO O 2p state under UV excitation), or defect-induced states near the Fermi level^38^ when hybridizing with Cu (Supplementary Fig. 13a). Furthermore, in the CZA catalyst, Cu LSPR-induced hot electrons could facilitate CO_2_ activation/dissociation at Cu surface sites (Fig. 3), accounting for the improved CO-production upon Cu excitation (CO mainly interact with Cu based on Fig. 3 and DRIFTS results).

Weakening of the CO_2_ C–O bond, proposed to occur as a result of filling of C–O antibonding orbitals by electrons from the substrate, could also potentially activate CO_2_ towards hydrogenation to formate, HCOO*. Examining the DFT-calculated orbital levels for the “formate radical” in Fig. 3 shows the singly occupied orbital associated with the unpaired electron localized on O, which would be associated with binding of the intermediate with the substrate. This orbital level at 7.26 eV overlaps well with the ZnO valence band, implying preferential adsorption of HCOO* at ZnO surface sites. However, the lowest lying fully vacant HCOO* orbital, corresponding to the HCOO* C–O π* antibonding orbital at 1.76 eV, overlaps well with the Cu 3*d* band. Any further hydrogenation of the formate intermediate must come at the expense of the C=O π bond, hence it is possible that filling of the C=O π* antibonding orbital by electrons originating from the Cu 3*d* band could facilitate formate hydrogenation. This corroborates the DRIFTS results which suggest that Cu plays a key role in catalyzing this process at the Cu/ZnO interface. Subsequent hydrogenation of HCOO* will lead to the formation of HCOOH*^8^. A weak physisorption of HCOOH* on Cu surface (BE_HCOOH*_ = −0.22 eV on Cu (111)), as reported by Grabow et al.^39^, could be implied. However, again in common with CO_2_, the lowest unoccupied molecular orbital (LUMO) of HCOOH* (C=O π* antibonding orbital), is well-aligned with the proposed level of the ZnO dominant excited state under UV excitation. This opens the possibility for activation of the HCOOH* intermediate by filling the antibonding orbital with electrons originating from excitation of dominant ZnO O 2p states. Accordingly, HCOO* conversion could be facilitated as the reverse reaction HCOO* + H* → HCOOH* is suppressed^8^. Afterwards, an increased interaction between Cu and H_2_COOH*, H_2_CO*, and H_3_CO* species are suggested with their first vacant orbitals well-aligned with the Cu 3*d* valence band, thus enabling the further hydrogenation to MeOH at near-Cu sites. The result further supports the hypothesis for an interfacial Cu/ZnO adsorption site being key to MeOH synthesis, with different elementary reaction processes being promoted by destabilisation of adsorbate bonds by electronic injection into vacant adsorbate antibonding orbitals from states associated with either ZnO or Cu.

### Light-induced Cu/ZnO state variations

**Fig. 4:**
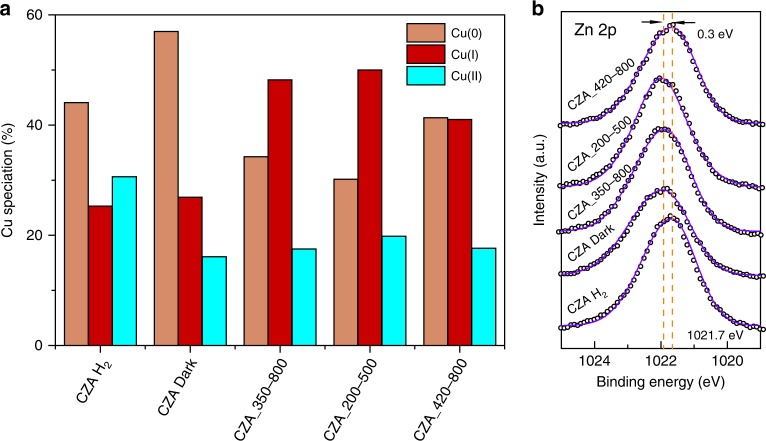
Copper and zinc states following different conditions. **a** Cu species distribution for reduced CZA (CZA H_2_) and CZA following reaction under different irradiation conditions (non-illuminated (CZA Dark), illuminated using different spectral range—350–800 nm, 200–500 nm, 420–800 nm). Data extracted from deconvoluted Cu LMM and Cu 2p spectra (see Supplementary Fig. 13 for details); **b** fitted Zn 2p spectra results for reduced CZA catalyst and spent catalysts under different irradiation conditions.

Under reaction conditions, the Cu oxidation state is influenced by the varied surface population of O* and H* species resulting from C–O bond breakage of the CO_2_ adsorbate and H_2_ dissociation, respectively. Auger spectroscopy and XPS were conducted to determine the Cu species distribution of fresh and spent CZA, which were tested under different irradiation conditions (herein referred to as CZA_350-800, CZA_200-500 and CZA_420-800, respectively). The result is shown in Fig. 4a. The dark reaction further reduced Cu(II) to Cu(0) (relative to the pre-reduced CZA) while retaining a similar Cu(I) content. When light was introduced during the reaction, the Cu(I) was enriched (irrespective of the spectral range) and the Cu(I)/Cu(0) ratio increased accordingly. This observation can be attributed to the promotion of dissociative CO_2_ chemisorption, leading to the formation of CO and Cu oxidation. A relatively lower Cu(I)/Cu(0) value (close to unity) for CZA_420-800 (Supplementary Fig. 14e) could be attributed to the Cu LSPR-triggered Cu–O weakening^13^ together with facilitated H_2_ dissociation on the Cu surface^40^, leading to a higher Cu(0) surface concentration.

The variation in binding energy (BE) for Zn 2p (Fig. 4b) is taken as an indicator of the overall electron exchange in the ZnO lattice under different reaction conditions. Upon careful inspection, it can be seen that Zn 2p BE of CZA_420-800 is similar to that of reduced catalyst (CZA H_2_), but lower (by ~0.3 eV) than that of spent catalysts under dark, under irradiation of 200–500 nm, and under irradiation of 350–800 nm. These indicate that for the latter three reaction conditions, a net electron transfer from ZnO to Cu could occur, while under visible light (420–800 nm), the excited Cu state could inhibit this process.

**Fig. 5:**
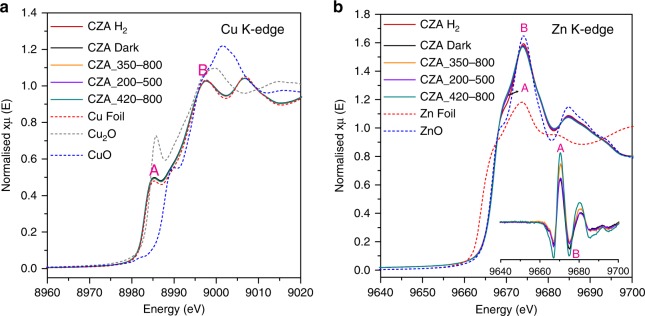
Local environment of Cu and ZnO following different conditions. **a** Cu K-edge and **b** Zn K-edge after reduction and reaction under dark or irradiation with different spectral ranges (350–800 nm, 200–500 nm, 420–800 nm). Inset: difference spectra obtained by subtracting the spectrum of reduced catalyst (CZA H_2_). Feature A and B indicate the low-energy shoulder and main absorption peak, respectively, in Cu and Zn K edge.

To gain insights into the local structure of Cu and ZnO, X-ray absorption was conducted at the Cu and Zn K-edges (Fig. 5, more details in Supplementary Fig. 15) on samples post reaction under differing irradiation conditions. Surprisingly, no sign of Cu oxide was detected and the X-ray absorption near edge structure (XANES) region of the X-ray absorption spectra exhibited identical spectroscopic features in all treated samples similar to that of the reference Cu foil, with a shoulder on the main edge (Feature A at 8981 eV) and a main absorption peak (Feature B at 8993 eV) ^41^, which appears to contradict the XPS observations. This could be explained by the difference of detection depth of the two characterization techniques, where XAS is a bulk characterisation technique giving average information for the whole sample and XPS is sensitive to the outermost surface atoms (<10 nm).

The Zn K-edge XANES spectra indicate that the overall oxidation state of ZnO in the reduced and spent catalysts did not change compared to the commercial ZnO reference, as there is no relative shift in the adsorption edge. The Zn K-edge spectrum is characterized by a main absorption peak (B, 1s → Zn 4p-O 2p hybridized states) located at 9669 K eV, a low-energy shoulder (A, 1s → Zn 4sp-O 2p hybridized states) at 9663 eV^41^. When subtracted with respect to the reduced CZA catalyst (inset of Fig. 5b), we detect an increase in the intensity of feature A, and a decrease in the intensity of main absorption peak B in CZA Dark.

These variations, according to the study of Garcia et al.^42^, could be due to either the change in coordination number or degree of occupation of outer Zn orbitals (via charge transfer). The former was excluded as the change in Zn coordination number was negligible in Supplementary Table 3. The change of Zn electronic states was therefore correlated to the change of states of neighbouring O atoms. Oxygen defects have been reported as important CO_2_ anchoring sites under reactive condition. As indicated in O1s spectra of XPS results (Supplementary Fig. 13a and Supplementary Note 7), the oxygen vacancy (O_v_) variation coincides with the changing pattern of feature A intensity, suggesting that the hybridization of Zn 4sp-O 2p was weakened at oxygen deficient sites. This change is believed to be related to the enhanced supply of H* from Cu to ZnO upon Cu excitation.

### Proposed mechanism

Vibrational spectra and valance band (VB)/DFT results show that, for MeOH synthesis over the Cu-ZnO hybrid, a defective ZnO surface (vacant oxygen sites with trapped electrons^43^, see Supplementary Fig. 13) provides the primary active sites for CO_2_ chemisorption via the form of CO_3_*/HCO_3_*/HCOO* species. Incorporating the Cu contributes to the subsequent hydrogenation processes, which is attributed to the promotional effect of the Cu–ZnO interface and Cu-stimulated H* supply. The active sites suggested by the molecular orbital energy levels and valence band electronic states for key intermediate species corroborate well with the in situ DRIFTS experiments, providing evidence for the Cu-ZnO interfacial sites being the main reaction sites for MeOH synthesis.

The distinct wavelength-sensitive photo-enhancement is ascribed to product-specific active sites (as also revealed in MeOH synthesis over the Pd/ZnO system^44^) on the CZA catalyst where CO production was photo-accelerated on the Cu surface with the assistance of electrons generated from Cu LSPR or ZnO bandgap excitation. In contrast, MeOH-relevant intermediate reactions were exclusively promoted through dual photo-excitation at the Cu-ZnO interfacial perimeter. HCOO*-to-H_3_CO* conversion represents the key challenge in promoting MeOH production over the CZA catalyst from our DRIFTS observation and the theoretical study from Kattel et al.^8^. We concluded that there is a synergy between Cu and ZnO for the synthesis of MeOH under UV-Vis light irradiation. When Cu alone was excited, the H* supply could be improved with LSPR-induced H_2_ cleavage on Cu and its migration to aid the formate hydrogenation step (reflected by a small increase in MeOH yield in Fig. 1e). When ZnO alone was excited, electron transfer to the Cu was highly favourable so no influence was observed on MeOH yield. Upon the dual excitation of Cu and ZnO, net electron transfer from the ZnO to Cu was attenuated and both H_2_ molecules and HCOO*-conversion were stimulated by light-generated electrons on the Cu and ZnO surfaces, respectively.

**Fig. 6:**
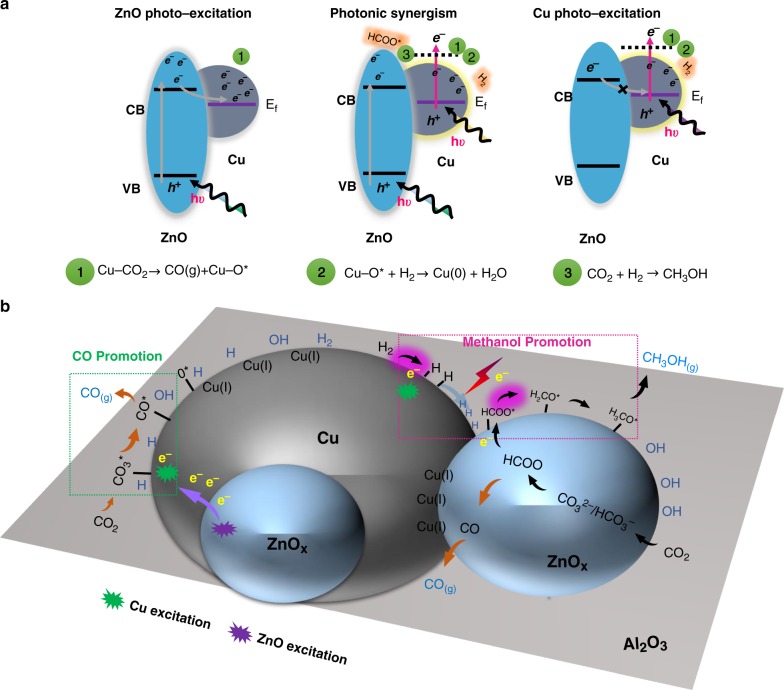
Proposed light-assisted CO_2_ hydrogenation mechanism. **a** Electron transfer between Cu and ZnO under irradiation by different light spectral ranges. The equations below the schematics indicate the potential reactions which could be improved when interacting with certain light-generated electrons. The potential electron acceptors are highlighted. **b** The light-aided CO_2_ hydrogenation reaction over Cu/ZnO/Al_2_O_3_ system. Pathways for CO (chemisorbed CO_2_ dissociation or ZnO–HCOO* decomposition) and methanol production (Cu–ZnO interface mediated processes) are illustrated with black and orange arrows, respectively. A dynamic population of species on Cu surface (Cu(I), H*, CO_3_*, CO*), ZnO surface (OH*, CO_3_*/HCO_3_*, HCOO*) and Cu–ZnO interface perimeter (HCOO*, H_2_CO, H_3_CO*) under reactive condition are envisioned. The electrons transferred to Cu surface promote CO_2_ dissociation to CO (green dotted rectangle), while the light-mediated methanol production is attributed to the promoted H*-supply and HCOO*-hydrogenation (glowing dark arrow) at the Cu–ZnO interface perimeter (red dotted rectangle) under dual excitation of Cu and ZnO.

Electron excitation and transfer under different irradiation conditions (Fig. 6a, see also Supplementary Note 8) is considered to be the principal driving force for the observed alterations in the Cu oxidation state, oxygen vacancy levels in ZnO, and CO_2_ hydrogenation performance. Cu-ZnO interplay was promoted during CO_2_ hydrogenation under dark condition as supported by the HRTEM images (Supplementary Fig. 1) and a positive BE shift in Zn 2p spectra (Fig. 4b). Under 200–500 nm irradiation, the ZnO excitation could promote electron transfer from the ZnO to Cu, which contributed to CO production and resulted in additional Cu(I)-O species (50% vs. 28% in dark) (Eq. (1) in Fig. 6a). Under 420–800 nm irradiation, only the Cu LSPR was excited; the generated hot electrons could have activated adsorbed H_2_ molecules, which triggered Cu(I)-O reduction (Eq. (2) in Fig. 6a) and oxygen defect creation in ZnO. Regardless, more Cu(I)-O (40%) was formed compared to that under the dark condition (28%). All of the aforementioned processes are likely to occur under 350–800 nm irradiation, whereby mediated Cu (with Cu(I) ratio of 48%) and ZnO states were finally established after reaching the charge equilibrium state. From XANES observations, we showed that the local electronic structures of ZnO were also affected by the population of adsorbates (namely H*) on the catalyst surface under different reaction conditions. In essence, full spectrum irradiation favours both H_2_ cleavage (Cu excitation) and formate conversion (ZnO excitation, potentially via facilitating HCOOH* activation according to Fig. 3), which greatly accelerated MeOH production (equation (3) in Fig. 6a) as supported by the time-resolved DRIFTS in Supplementary Fig. 16a.

## Discussion

We have studied CO_2_ hydrogenation by the industrially-relevant CZA catalyst under simultaneous heat and light activation. Notably, MeOH production was promoted (by >30%) under UV-Vis irradiation (350–800 nm). The photothermal catalytic system demonstrated the capability to achieve a more efficient and selective production of MeOH at a temperature that is 50 °C lower than that of the thermal catalytic system alone. In contrast, CO production was boosted under irradiation, irrespective of the spectral range in the present study. Post-reaction structural and electronic properties (XPS and XAS) in conjunction with catalytic reaction mechanistic studies (DRIFTS) suggested that photo-generated electrons and their subsequent interfacial transfer were responsible for the simultaneous transformations in surface chemistry and catalytic reactions. Electrons from either ZnO band gap excitation (UV irradiation) or Cu plasmon excitation (visible light irradiation) assisted direct CO_2_ dissociation to CO followed by Cu(I)-O formation on the Cu surface. In addition, high energy electrons from the Cu LSPR facilitated Cu(I)-O reduction through improved H_2_ cleavage on Cu surface and its migration to ZnO. Upon concurrent excitation of Cu and ZnO, net electron transfer was attenuated and the sluggish surface reactions—HCOO* hydrogenation and H_2_ cleavage—were accelerated on the ZnO and Cu, respectively. This led to enhanced MeOH production at the Cu-ZnO interfacial perimeter. The study illustrates the synergy between pivotal adsorbed intermediate species (e.g., HCOO*, H*) at the interfacial perimeter of Cu-ZnO, and provides understanding on the light-heat synergism in site-specific catalytic systems; more generally it gives insight into the relative roles of ZnO and Cu in Cu/ZnO catalysed MeOH synthesis.

## Methods

### Materials

Chemicals were used as supplied: copper (II) nitrate (Cu(NO_3_)_2∙_3H_2_O, Sigma-Aldrich); zinc nitrate (Zn(NO_3_)_2_.6H_2_O, Sigma-Aldrich); aluminium nitrate (Al(NO_3_)_3∙_9H_2_O, Sigma-Aldrich); sodium carbonate (Na_2_CO_3_, Sigma-Aldrich).

### Catalyst synthesis

All metal oxides were prepared via a conventional co-precipitation method. Typically, 14 ml 0.5 M Cu(NO_3_)_2_, 4 ml 0.5 M Zn(NO_3_)_2_, and 2 ml 0.5 M Al(NO_3_)_3_ were added to a 50 ml beaker to give a mixed solution with a molar ratio of Cu:Zn:Al=7:2:1. After stirring at 65 °C for 20 min in an oil bath, 10 ml 1.2 M Na_2_CO_3_ solution was added dropwise as the precipitant with the solution then stirred for another 10 mins. The precipitates were aged (>60 min), washed, filtered and dried overnight (>80 °C). The obtained precursor was ground and calcined at 250 °C for 3 h (5 °C min^−1^ ramping rate, 30 ml min^−1^ air flow). Reduction was conducted either in a tube furnace (ex situ at 250 °C for 3 h under 10% H_2_/N_2_) or a Harrick reactor (in situ at 350 °C for 1 h under 10 ml min^−1^ H_2_). The reduced Cu/ZnO/Al_2_O_3_ catalyst is denoted as CZA. Catalysts containing Cu+Al, Cu+Zn, and Zn+Al were synthesised using the same procedure and the same metal ratio (Cu:Zn:Al = 7:0:1, 7:2:0, and 0:2:1, respectively) as controls. The reduced Cu/Al_2_O_3_, Cu/ZnO, and ZnO/Al_2_O_3_ catalysts are denoted as CA, CZ, and ZA, respectively.

### Catalyst characterisation

High angle annular dark field-scanning transmission electron microscopy (HAADF-STEM) and Energy Dispersive X-ray (EDX) spectroscopy of the CZA and spent CZA catalysts were conducted on a JEOL JEM-ARM200F operating at 200 kV. The crystal phase of the prepared catalysts was analysed using a PANalytical Xpert Multipurpose X-ray Diffraction (XRD) System. UV-Vis spectra were recorded with a Shimadzu UV-3600 UV-Vis-NIR Spectrophotometer using BaSO_4_ as the reference. Cu dispersion, temperature-programmed H_2_ reduction (H_2_-TPR), and temperature-programmed CO_2_ desorption (CO_2_-TPD) were conducted on an AutoChem II 2920 system. For Cu dispersion analysis, Cu oxide was first reduced to Cu metal in a gas flow mixture of 10% H_2_-Ar (ramping temperature to 673 °C at 10 °C min^−1^) during first H_2_-TPR test. Then purged with Ar for 1 h after cooling to 50 °C. Oxidation of Cu to Cu_2_O was assessed by N_2_O treatment (at 50 °C) with a second H_2_-TPR on the formed Cu_2_O then conducted (same procedure with first H_2_-TPR applied). Cu dispersion was derived from the H_2_ uptake (*Q*) in two TPR curves: Cu dispersion = 2*Q* (2^nd^ TPR)/*Q* (1^st^ TPR).

XAFS measurements were performed on the B18 beamline at the Diamond Light Source, Didcot, UK. Measurements were performed in transmission mode using a QEXAFS setup with fast-scanning Si (111) double crystal monochromator. XAFS spectra were acquired in 60 s and averaged over 3 scans, concurrently with the appropriate foil as reference and for simultaneous reference data collection. The spent catalysts were scanned at the Cu K edge (kmax = 12 Å) and Zn K edge (kmax = 9.5 Å). The kmax for Zn K edge was reduced due to the close proximity of the Cu K edge. Samples of spent catalysts were sealed in the Harrick DRIFTS cell under Ar post reaction with the use of isolation valves and were placed into a glove box for sample protection. No samples were exposed to air during the entire course of sample analysis to avoid sample oxidation from atmospheric oxygen. All spent catalysts were purged with Ar for 10 min during the cooling phase after 3 h of reaction (3 h of dark reaction or 1 h of dark reaction with 2 h of light irradiation) and were transferred out of the reactor immediately into the glove box once the reactor reached room temperature (typically within 10 min of Ar flow). Spent catalysts were mixed with an appropriate amount of cellulose for dilution and were made into pellets and sealed in an Ar-contained gas cell for XAFS measurements. XAFS data was processed using Athena and Artemis software within the Demeter package.

XPS measurements were performed with a Kratos Axis Supra equipped with an Al Kα source (1486.68 eV, 150 W, 10 mA × 15 kV). All XPS spectra were normalised to the C1s peak = 285.0 eV for adventitious carbon. The spent catalysts samples were treated the same way as for XAFS measurements (without exposure to air), without dilution with cellulose and pelleting. Data analysis was performed using the Avantage software. A smart-Shirley background was applied to all spectra.

### Photothermal CO_2_ hydrogenation

Photothermal CO_2_ hydrogenation experiments were performed using a Harrick flow reactor system at varying pressures (1–21 bar) as shown in Supplementary Fig. 17. The reactor system comprised: (i) a LX300F Xe illuminator (Perkin Elmer/ILC Technologies) mounted in an Eagle R300-3J lamp housing; and (ii) a stainless-steel Harrick reactor (HVC-MRA-5, Harrick’s Scientific, USA) with 13 × 2 mm SiO_2_ window for irradiation from above and compensatory heating from below (heating cartridge and K-type thermocouple). The light source was placed 6 cm above the reactor window and was directed towards the reactor with a MR 60/90 light reflection unit. Additional control of the spectral region of light incident upon the reactor was provided by optional cold mirrors (range of 200–500 nm or 350–800 nm) and/or a Schott GG-420 long pass glass filter (visible light irradiation, >420 nm). The intensity of the light source was governed by the current output. The intensity of the different light sources incident upon the catalyst bed was controlled to be around 600 mV cm^−2^ (measured with a reference silicon solar cell and light intensity meter). The reactor temperature was controlled by a thermocouple and heating system connected to the Harrick reactor under both dark and light experiments. Prior to reaction, 30 mg of catalyst was reduced in situ at 350 °C (ramp rate 5 °C min^−1^) for 1 h under a 10 mL min^−1^ hydrogen flow. Subsequently, CO_2_ hydrogenation from 200 to 400 °C was performed with H_2_ + CO_2_ (with a ratio of H_2_: CO_2_ = 3.2:1) feedstock (flow rate = 20 mL min^−1^). At each temperature step, gas samples were collected after >30 min to allow the reaction to reach steady state. Samples from the reactor outlet were injected into a gas chromatograph (Shimadzu GC2010-Plus) equipped with a thermal conductivity detector (TCD) and flame ionization detector (FID), and a mass spectrometer to help identify the products. The stainless-steel gas line between the reactor and GC system was heated (at 130 °C) to avoid the condensation of the liquid products. Only CO and MeOH were detected as the products over CZA catalyst (methane production detected in vibrational spectra is below the detection limit of GC), the MeOH production performance was evaluated with MeOH space time yield (MeOH STY) and MeOH selectivity (Eqs. 1 and 2, respectively):

1 $${\mathrm{MeOH}}/{\mathrm{CO}}\,{\mathrm{STY}} = \frac{{F_{{\mathrm{tot.}}} \times R_{{\mathrm{CO}}/{\mathrm{MeOH}}} \times 10^{ - 3} \times 60}}{{V_{\mathrm{m}} \times m_{{\mathrm{cat.}}} \times 10^{ - 5}}}$$

2 $${\mathrm{MeOH}}\,{\mathrm{selectivity}} = \frac{{{\mathrm{MeOH}}\,{\mathrm{STY}}}}{{{\mathrm{MeOH}}\,{\mathrm{STY}} + {\mathrm{CO}}\,{\mathrm{STY}}}}$$

where, *F*_tot._ (ml min^−1^) is the total flow rate of outlet gas, *R*_CO/MeOH_ (%) is volume/flow ratio of MeOH/CO product calculated from TCD/FID peak signal using calibrated peak area-flow ratio relation curve. *V*_m_ (L mol^−1^) is the molar volume of gases. *m*_cat._ (mg) is the mass of catalyst (30 mg).

### In situ diffuse reflectance infrared Fourier transformed spectroscopy

In situ DRIFTS was performed using a Brüker VERTEX 70v FTIR spectrometer equipped with a liquid N_2_-cooled MIR source, KBr optics, and a RockSolid interferometer. The home-made DRIFTS set-up, including the provision of in situ irradiation (350–800 nm) during analysis, is depicted in Supplementary Fig. 18. To maintain consistency, irradiation of the DRIFTS sample was conducted using a Xe lamp as the light source and a Ø4 mm aluminium reflective collimator was installed inside the DRIFTS chamber. A 2 m custom made patch cable with Ø1000 µm, 0.50 numerical aperture, high hydroxide (OH), core step-index multimode fibre terminated by Sub-Miniature version A (SMA) mating sleeves was used to connect the Xe lamp (adapter used) and the collimator. For each analysis, ~30 mg of (diamond-diluted) catalyst (10 wt.% of catalyst) was placed in a commercial in situ DRIFTS cell [HVC-DRM-5, Harrick’s Scientific, USA] possessing ZnSe windows and equipped with an ohmic heating device. Cu-containing samples (CZA, CA) were diluted with inert diamond for receiving an ample reflectance signal amplitude (pure reduced Cu samples are black), then pre-treated by calcining at 400 °C in air to remove residual organic species, followed by in situ reduction in a Harrick® reaction cell at 350 °C in pure H_2_ (flow rate = 20 mL min^−1^) for 1 h. After cooling to 50 °C under N_2_, the system was pressurized to 15 bar with an elevated N_2_ flow rate (5–15 ml min^−1^) (in which the spectrum was taken as a background). In situ DRIFTS analysis was then performed for temperature programmed CO_2_ hydrogenation from 50–300 °C with a 3:1 ratio of H_2_:CO_2_ feedstock (total flow rate = 8 mL min^−1^). At each temperature step, the steady-state spectra and purged spectra (N_2_ purging for 1.5 h) were both recorded with 64 scans at 1 cm^−1^ resolution in the range of 600–4000 cm^−1^. For the light-assisted reaction, Xe lamp irradiation was directed into the reactor using the optic fibre and collimator (see scheme S2).

### Computational details

Gaussian 16 code^45^ was used for the calculation of orbital energy of free gas phase reactant, product and species. The PBE exchange–correlation functional was used throughout^46^, and a split-valence triple-ζ quality Pople basis set was applied, with a single polarisation function included (6–311 G*)^47,48^. DFT calculations were performed for each of the free gas phase reactant, product and intermediate species using the Gaussian 16 code. All structures were optimised such that all forces on atoms were converged to within 0.0003 *E*_h_/*a*_0_. The PBE exchange–correlation functional was used throughout, and a split-valence triple-ζ quality Pople basis set was applied, with a single polarisation function included (6–311 G*). Details can be found in supporting information.

In order to afford comparison with the CZA VB spectra, the calculated projected density of states (PDOS) is presented for bulk Cu in Fig. 3. Plane-wave DFT as implemented in the VASP v5.4.4 code^49,50^ using a plane-wave cut-off of 450 eV and the PBE^46^ exchange–correlation functional. Optimisation of the bulk FCC Cu lattice parameter was performed by fitting to the Birch–Murnaghan equation of state before performing the PDOS calculation, obtaining a lattice parameter of 3.63 A for a cubic close packed periodically repeating cell consisting of four Cu atoms. The Monkhorst-Pack k-point sampling scheme^51^ was applied, using a dense 24 × 24 × 24 k-point sampling mesh to enable accurate calculation of the PDOS.

## Supplementary information

Supplementary information

Peer Review

## Data Availability

The data that support the plots in this paper and the other findings of this study are available from the corresponding authors on reasonable request.
