# Supplementary material for: Synergistic ultraviolet and visible light photo-activation enables intensified low-temperature methanol synthesis over copper/zinc oxide/alumina
Source: Nat Commun. 2020 Mar 31;11:1615. doi: 10.1038/s41467-020-15445-z (PMC7109065; doi:10.1038/s41467-020-15445-z)
Supplement: Supplementary file 1 — Supplementary information [file 41467_2020_15445_MOESM1_ESM.pdf]

Supporting Information

Synergistic ultraviolet and visible light photo-activation enables intensified low-temperature methanol synthesis over copper/zinc oxide/alumina

Xie *et al.*

Supplementary Figs.1-18: Page 1-18

Supplementary Tables 1-3: Page 19-21

Supplementary Notes 1-8: Page 22-30

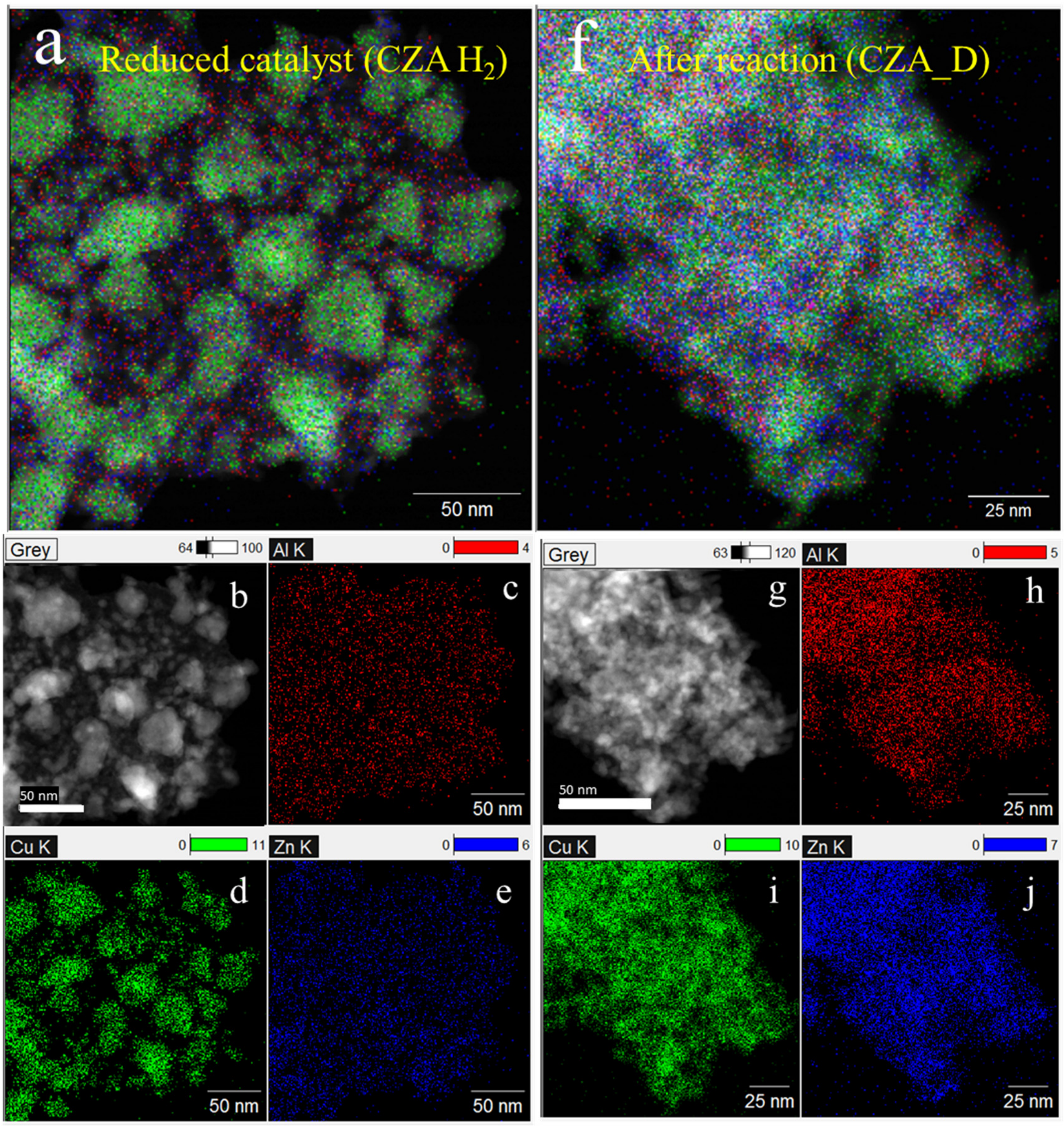

Supplementary Fig. 1. STEM-EDX elemental mapping micrographs. (**a-e**) reduced catalyst (CZA H_2), and (**f-j**) tested catalyst under dark (CZA Dark). Reaction conditions: CO_2:H_2 = 1:3.2; GHSV = 8758 h^{-1}; Al = red, Cu = green, Zn = blue. STEM-EDX of the spent catalyst shows redispersion of the Cu nanoparticles, implying an enhanced Cu-ZnO contact/interaction under reaction conditions. Collective surface plasmon resonances, which can be excited by light, was proven to exist in reduced and spent CZA catalysts by electron energy-loss spectroscopy (EELS) (Supplementary Figs. 4 and 5).

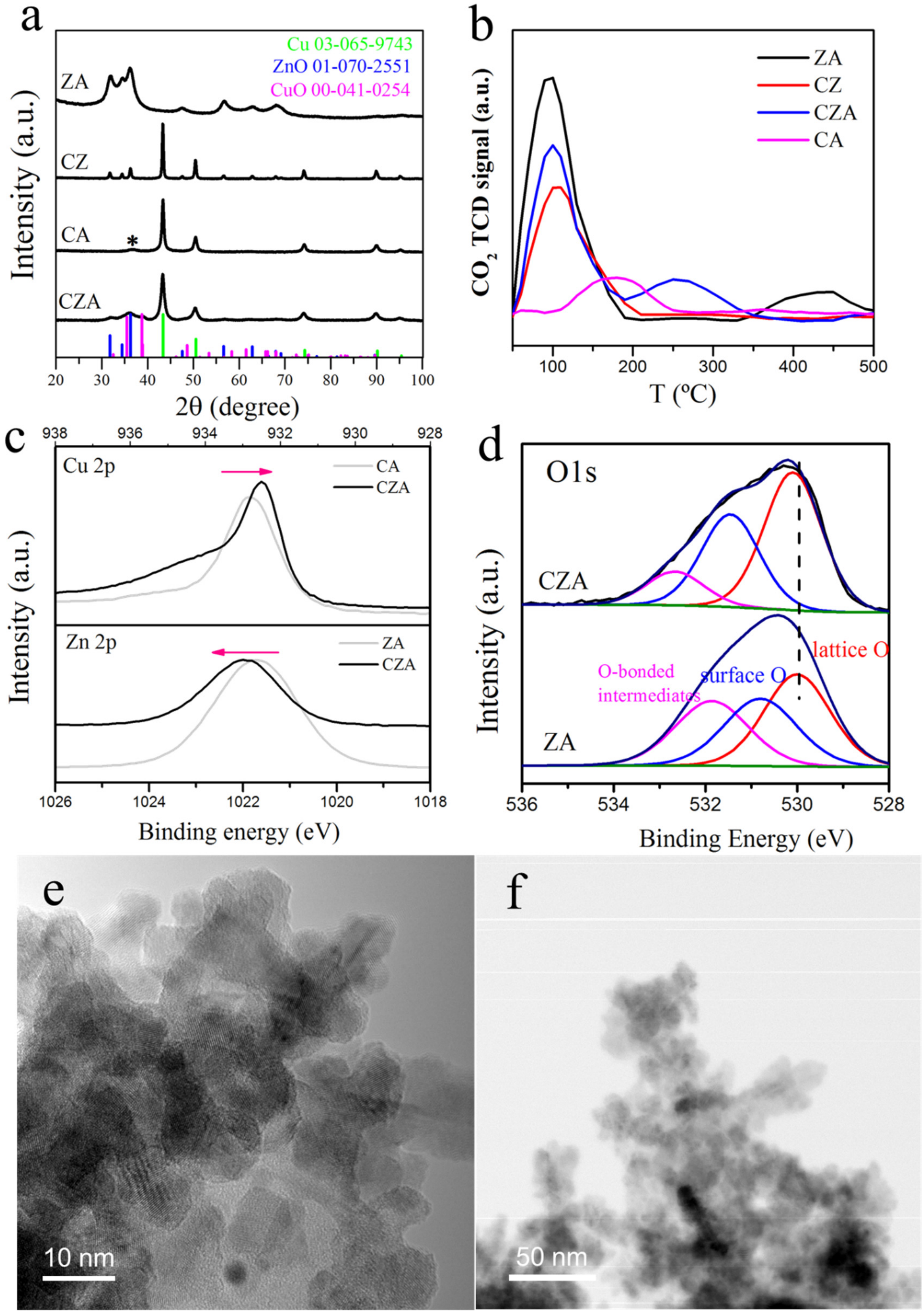

Supplementary Fig. 2. Supplementary characterisation results for catalysts. (**a**) X-ray diffraction (XRD) profiles; (**b**) CO_2 temperature programmed desorption (CO_2-TPD) profiles; (**c**) Cu 2p, Zn 2p and (**d**) O 1s X-ray photoelectron spectroscopy (XPS) spectra of reduced Cu/ZnO (CZ), ZnO/Al_2O_3 (ZA), Cu/Al_2O_3 (CA), and $Cu/ZnO/Al_2O_3$ (CZA) catalysts; (**e,f**) HR-TEM images of the reduced CA sample.

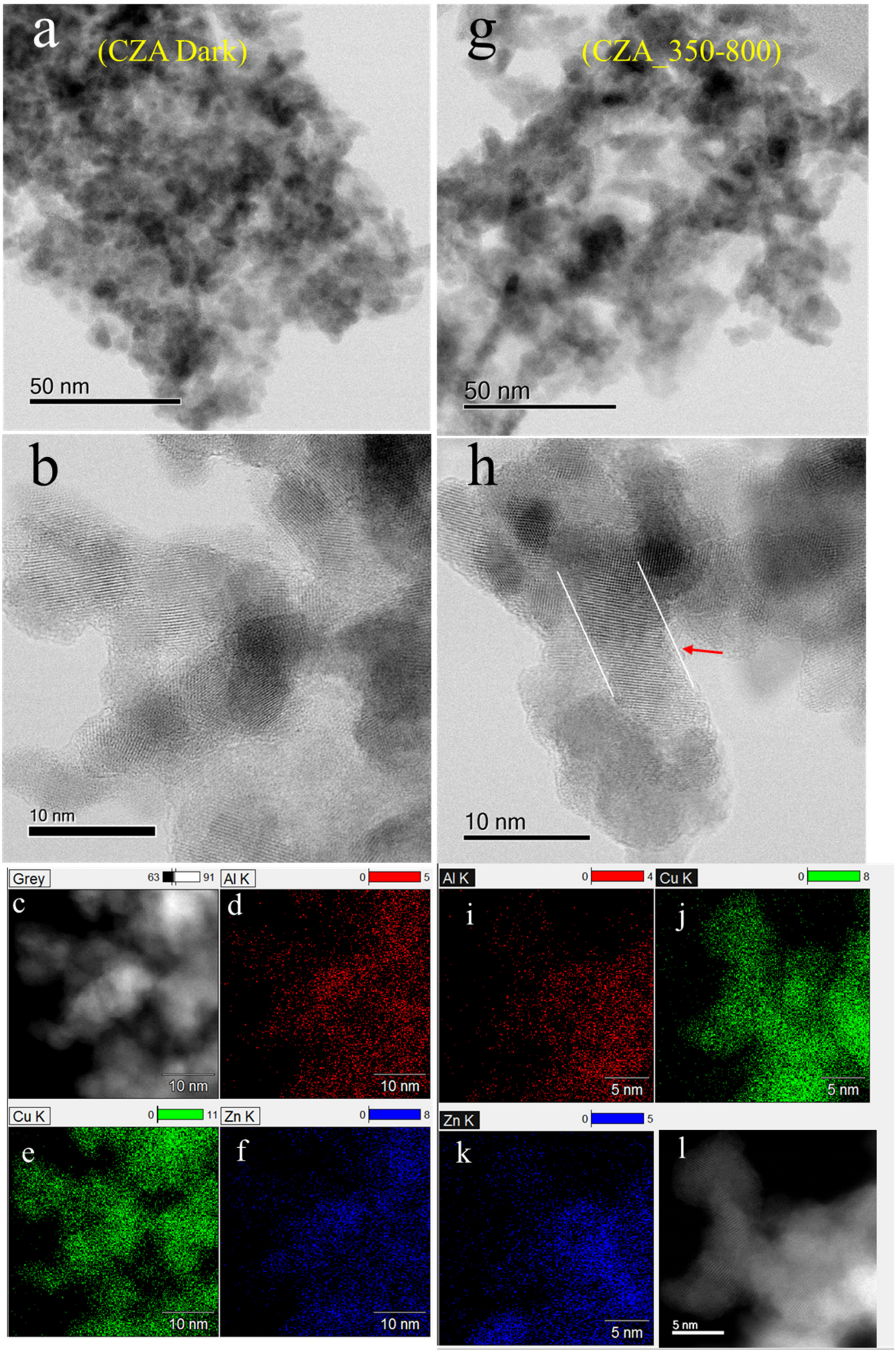

Supplementary Fig. 3. Morphology and microstructure comparison between CZA Dark (a-f) and CZA_350-800 (g-l). Reaction conditions: CO_2:H_2 = 1:3.2; GHSV = 8758 h^{-1}; P = 21 bar; light intensity = 600 mW cm^{-2} for CZA_350-800. Al = red, Cu = green, Zn = blue.

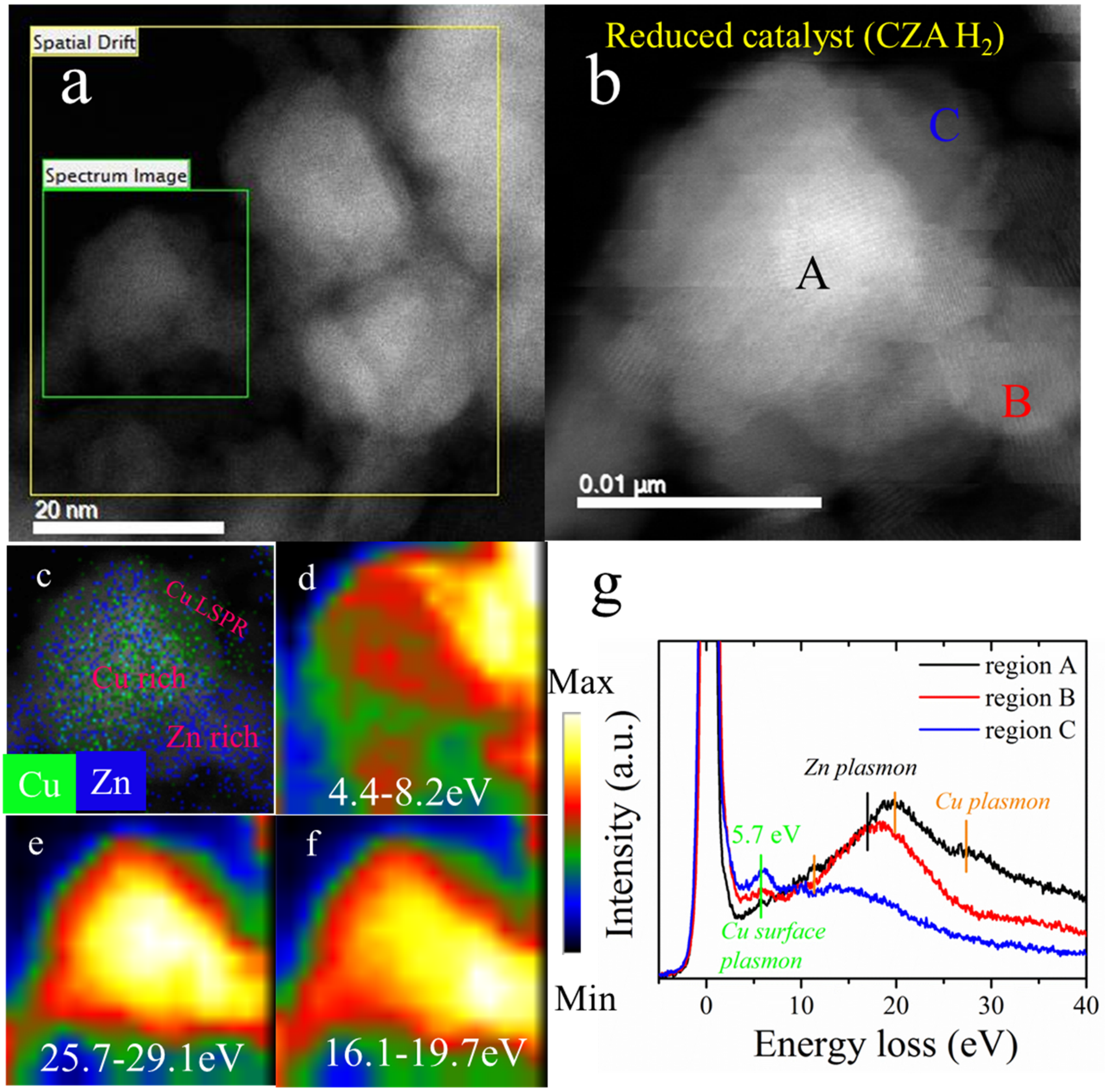

Supplementary Fig. 4. Selected-region plasmon resonance. HAADF pictures (**a, b**), element mapping picture (**c**), and selected-energy Electron Energy Loss Spectroscopy (EELS) mapping (**d-f**) and selected-region profiles (**g**) of CZA H_2.

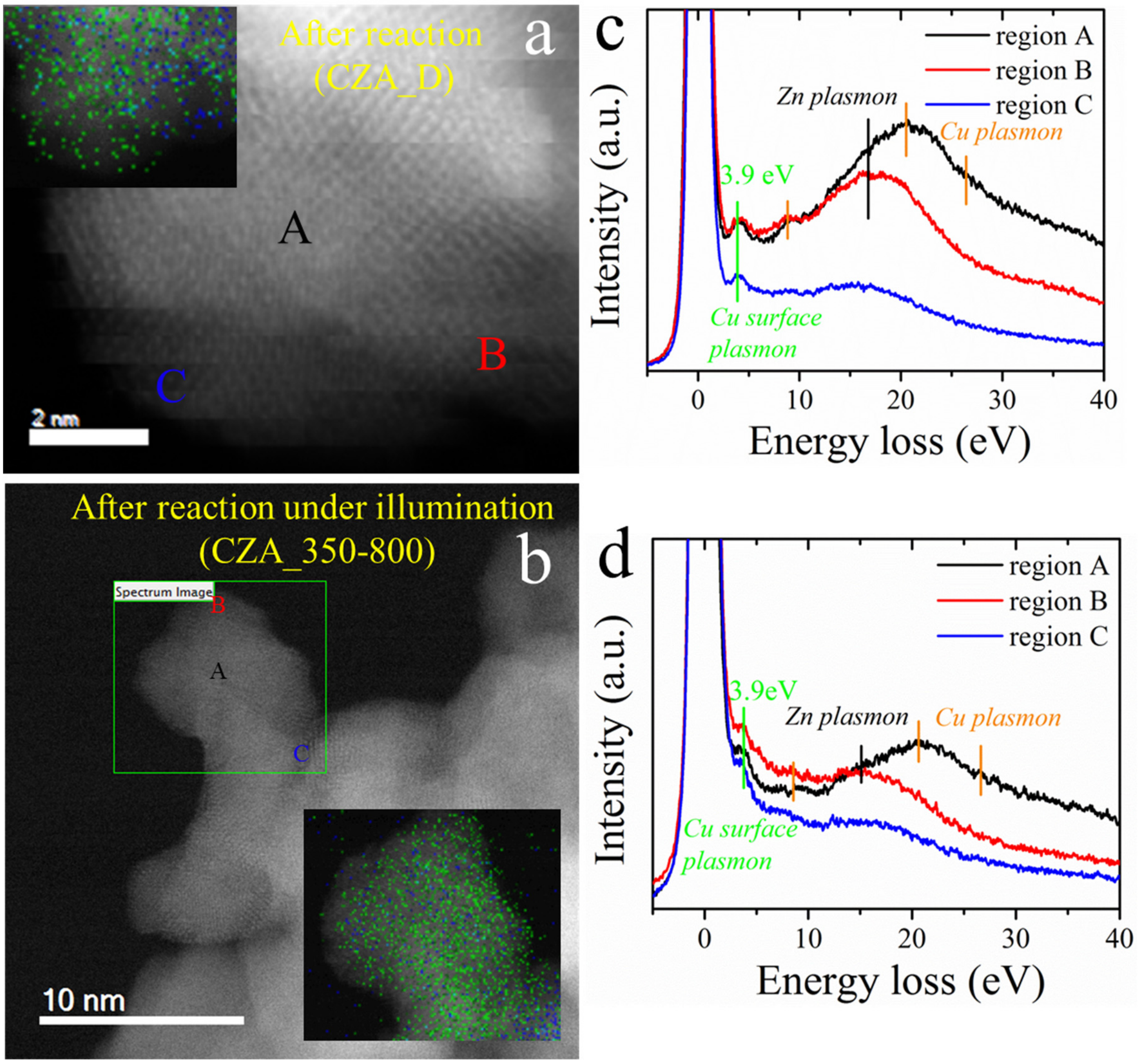

Supplementary Fig. 5. Selected-region plasmon resonance. HAADF pictures (**a, b**), and selected-region Electron Energy Loss Spectroscopy (EELS) profiles (**c, d**) of CZA Dark (**a, c**), and CZA_350-800 (**b, d**). Insets of (**a, b**) show the corresponding element mapping profiles. Low-energy electron energy loss spectroscopy (EELS) explicitly revealed the presence of Cu/Zn bulk plasmon and Cu surface plasmon resonance (at ~5.7 eV)[1], which was confirmed by the selected-energy EELS mapping profiles. There is a change in the position of Cu surface plasma peak (5.7 eV *vs.* 3.9 eV in Supplementary Fig. 4 and 5, respectively), which is correlated to the vibrations in localized chemical environment and size of Cu nanoparticles after reaction.

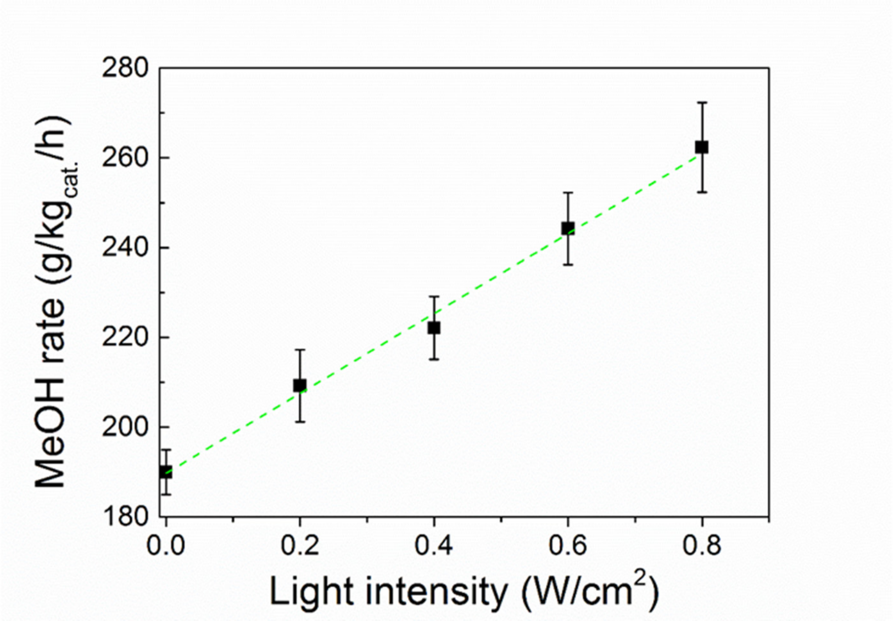

Supplementary Fig. 6. **Intensity-dependent photothermal MeOH production rate**. The dashed line shows a near-linear relation between rate and light intensity (R^2=0.994). Error bars indicate the deviation among several sampling (injection) at each light intensity.

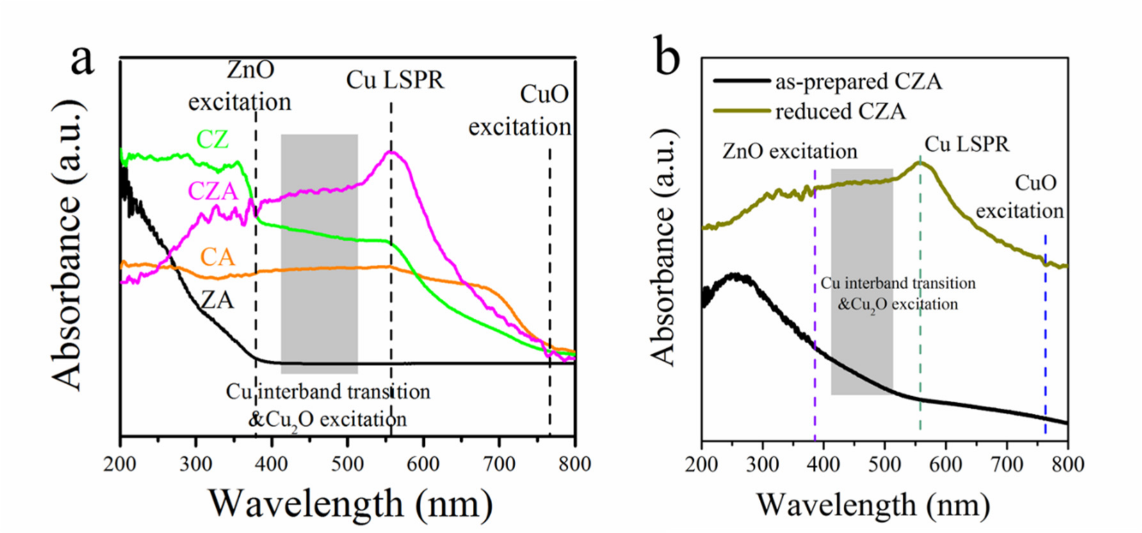

Supplementary Fig. 7. UV-vis spectra of (**a**) reduced CA, ZA, CZ, and CZA sample, and (**b**) comparison between as-prepared CZA and reduced CZA sample. UV-vis absorption spectra provide key information on the photo-excited regions for the different components. Light with energy E > 3.2 eV (wavelength λ< 390nm) is sufficient to activate ZnO via band-gap excitation (ZnO absorption in CZA is masked by the strong metallic Cu absorption). The Cu localized surface plasmon resonance (LSPR) is located at around 560 nm. A strong damping of Cu plasmon is observed in the Cu-based catalysts due to its resonance with the Cu interband transition region[2] (~590 nm, appears as a strong "background" absorption next to plasmon resonance peak). The absorption of CuO and Cu_2O (wavelengths of 826 nm and 516 nm, respectively) also resides on either side of the Cu LSPR peak.

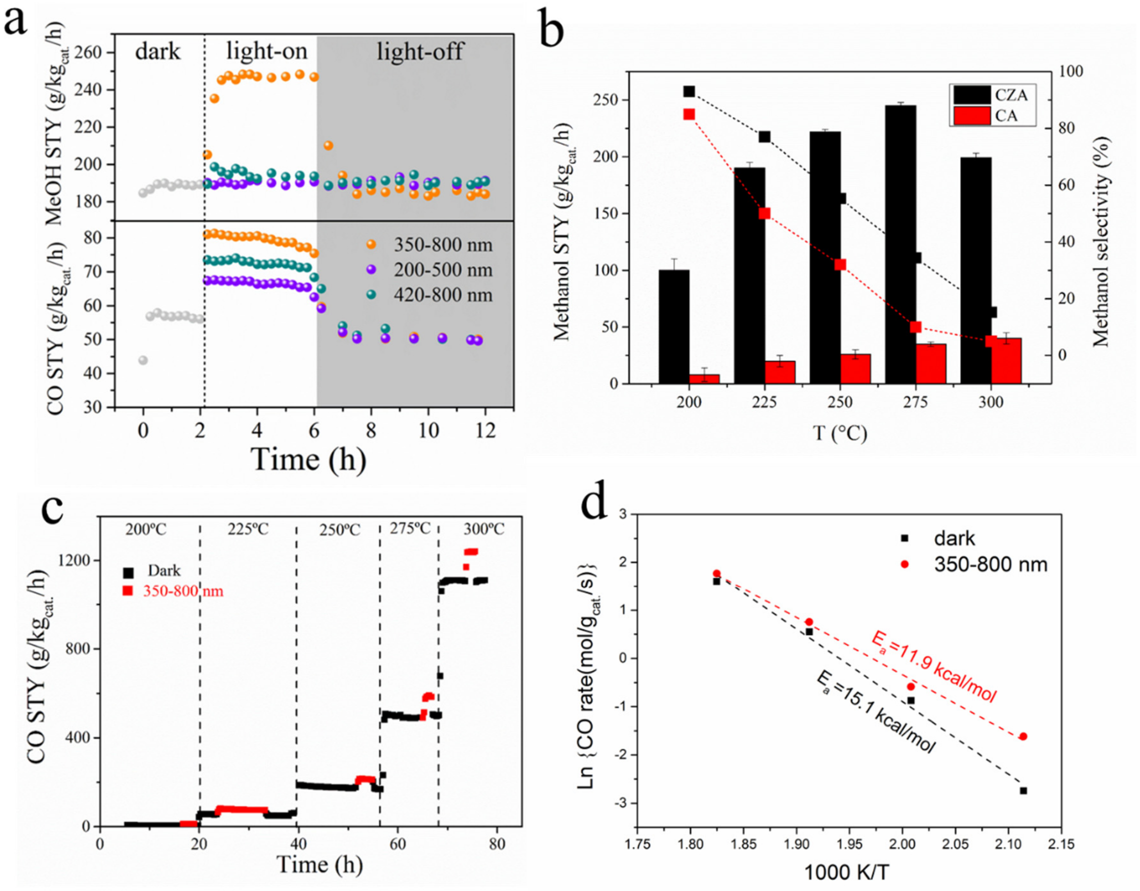

Supplementary Fig. 8. Supplementary performance results: (a) MeOH (top) and CO (bottom) STY at 225 ºC initially under no illumination (dark) followed by illumination (light-on) with one of three distinct wavelength ranges (350-800 nm, 420-800 nm, or 200-500 nm) followed by a return to non-illuminated conditions (light off); (**b**) MeOH space time yield (STY) and MeOH selectivity over a temperature range of 200-300 °C for CZA and CA. Error bars indicate the deviation among three independent experiments; (**c**) CO STY for CZA at different temperatures under non-illuminated (dark) and with 350-800 nm light illumination; and (**d**) Calculated activation energy for the RWGS reaction under dark and light conditions.

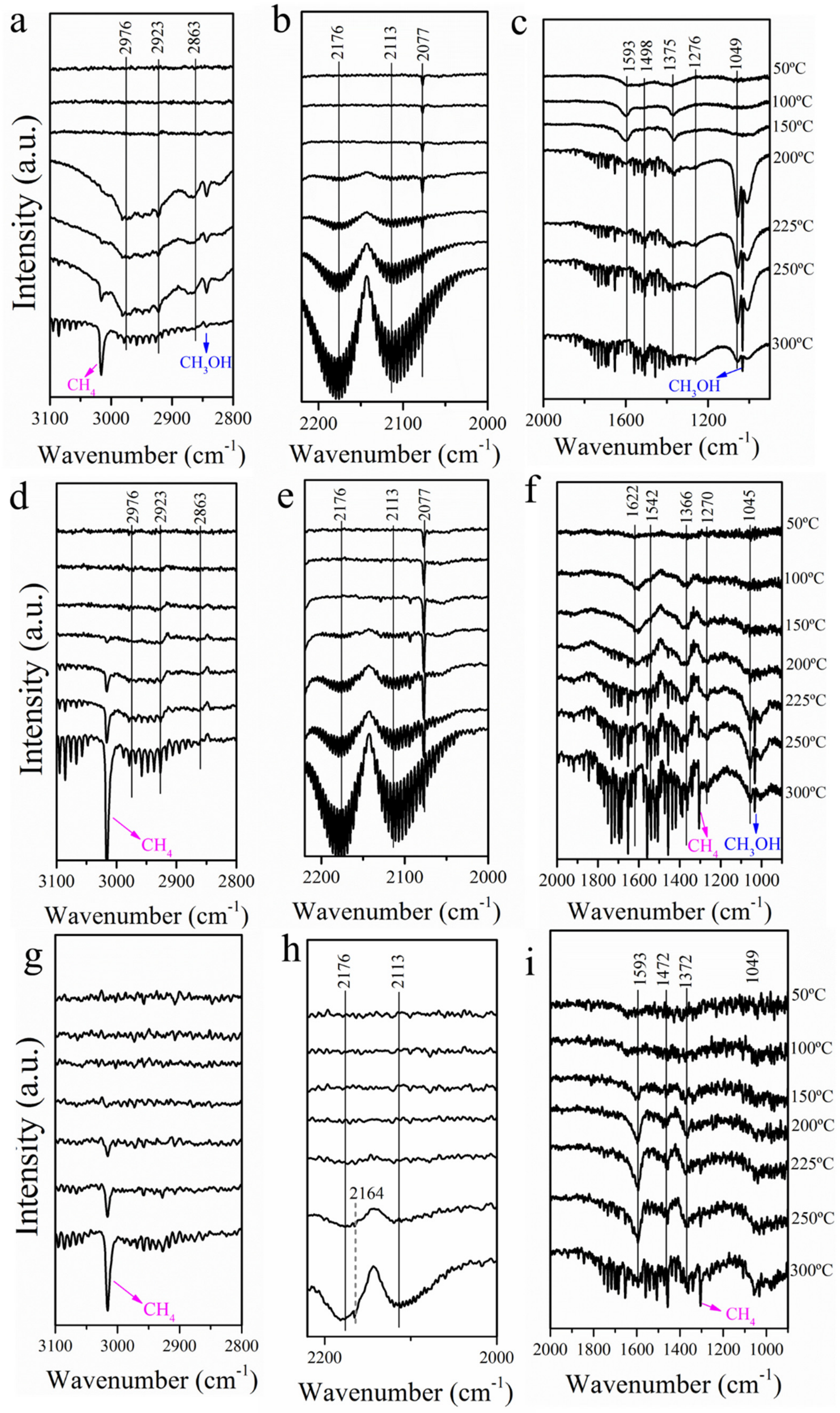

Supplementary Fig. 9. DRIFTS spectra of (**a-c**) CZA, (**d-f**) CA, and (**g-i**) ZA when exposed to CO_2 and H_2 atmosphere at 15 bar and 50-300 ºC.

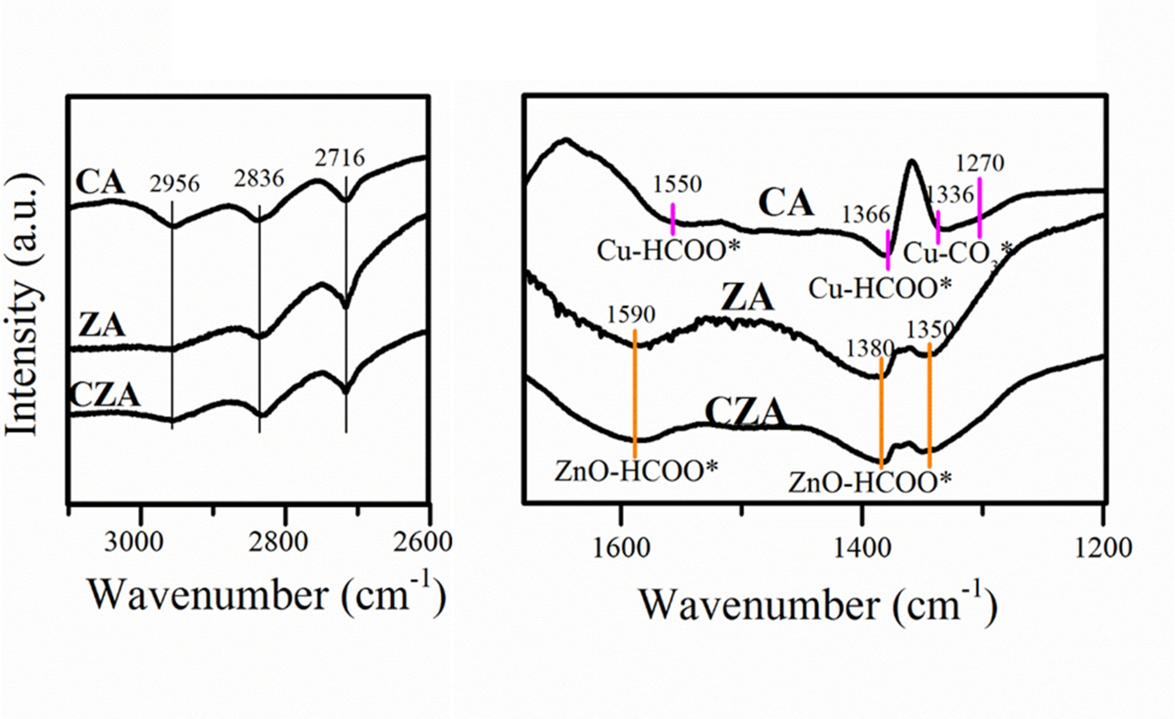

Supplementary Fig. 10. DRIFTS spectra of CA, ZA, and CZA sample adsorbed with formate species. Sample powder was immersed in sodium formate solution and then dried at 100 ºC for 1h before transferring to IR cell. The spectra are characteristic of two groups of signals: Cu-HCOO* with vibrational peak at 1550, and 1366 cm^{-1}, and ZnO-HCOO* with vibrational peak at 1590, 1380, and 1350 cm^{-1}.

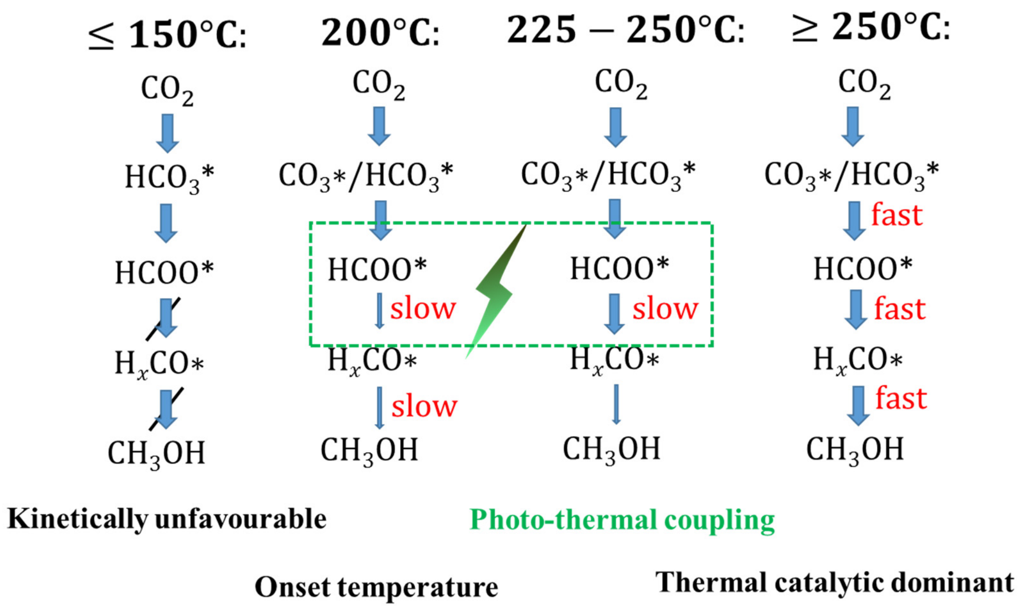

Supplementary Fig. 11. Intermediate-involved reactions under different temperatures. The reaction rate of each specific intermediary step was deduced from the residual species information reflected by DRIFTS spectra at a certain temperature and their subsequent cross-comparison.

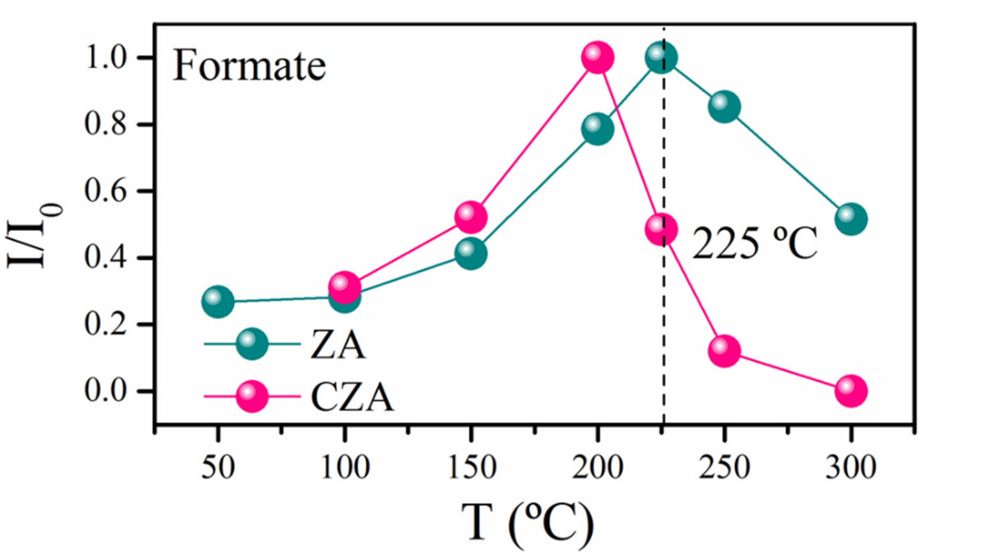

Supplementary Fig. 12. Normalized formate intensity comparison between CZA and ZA. Formate intensity value was normalized by the highest peak intensity from temperature-dependent DRIFTS spectra in Fig. 2b and Supplementary Fig. 9. Although with a same HCOO* vibrational mode (1593 cm^{-1}) in two catalysts, the conversion of formate was promoted with the presence of Cu-ZnO (CZA) interaction compared to ZA catalyst.

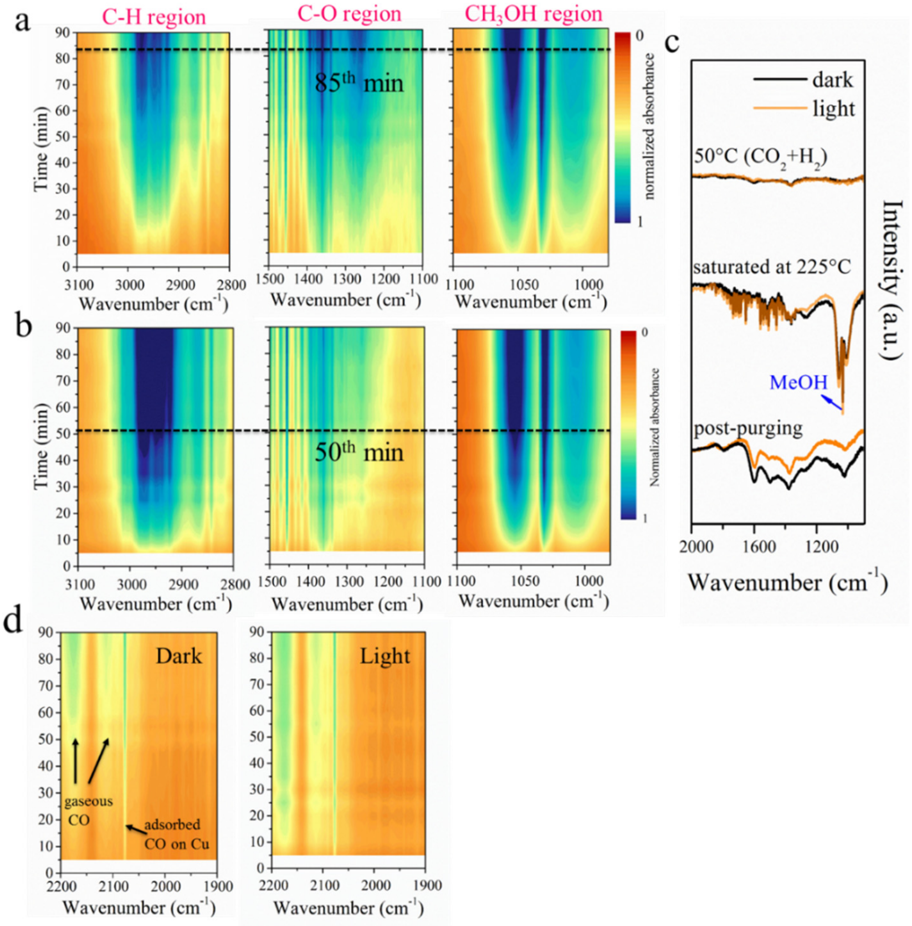

Supplementary Fig. 13. Kinetics difference detected *via* DRIFTS. Normalised transient spectrum of CZA recorded at 225 ºC in the first 90 mins (CO_2 + H_2 gas, the absorbance is normalized based on equilibrium-state spectrum at 90th min) under (**a**) dark; and (**b**) 350-800 nm light illumination conditions - three distinctive spectrum regions at 980-1100, 1100-1500, and 2800-3100 cm^{-1}, assigned to the representative vibrations of CH_3OH, O-C=O and C-H bonds, respectively, are present which reveal the varying tendency of products and intermediates in the first 90 mins (after the reaction temperature had reached 225 ºC); (**c**) spectra recorded at different time points during dark and light experiments; **(d)** time-resolved CO formation over CZA under non-illuminated (dark) and light illumination range of 350-800 nm, revealing the varying tendency of gaseous CO (2113, 2176 cm^{-1}) and adsorbed CO (2077 cm^{-1}) in the first 90 mins (after the reaction temperature had reached 225 ºC). It shows that, under light illumination, the formation of gaseous and adsorbed CO was accelerated, and more adsorbed CO was found to form on the Cu surface after reaction. Spectra depicting differences in the levels of accumulated surface intermediates and MeOH product during and after reaction, following reaction at 225 °C for 90 min and with consequent purging (to remove water) under dark and illuminated conditions are presented. The system reached steady state after ca. 85 min under the dark condition (Supplementary Fig. 13a); however, under light irradiation (350-800 nm) the surface reaction was accelerated with steady state attained in 50 min (Supplementary Fig. 13b). With reference to the steady-state DRIFTS spectra collected at 50 °C, a higher MeOH concentration during reaction and a lower concentration of surface intermediates are apparent with light irradiation at 225 °C (Supplementary Fig.13c), which suggests that formate hydrogenation step was accelerated and a "new" reaction equilibrium was accordingly achieved during light-assisted MeOH production. Similarly, CO formation was shown to be accelerated on the Cu surface under light irradiation (Supplementary Fig. 13d).

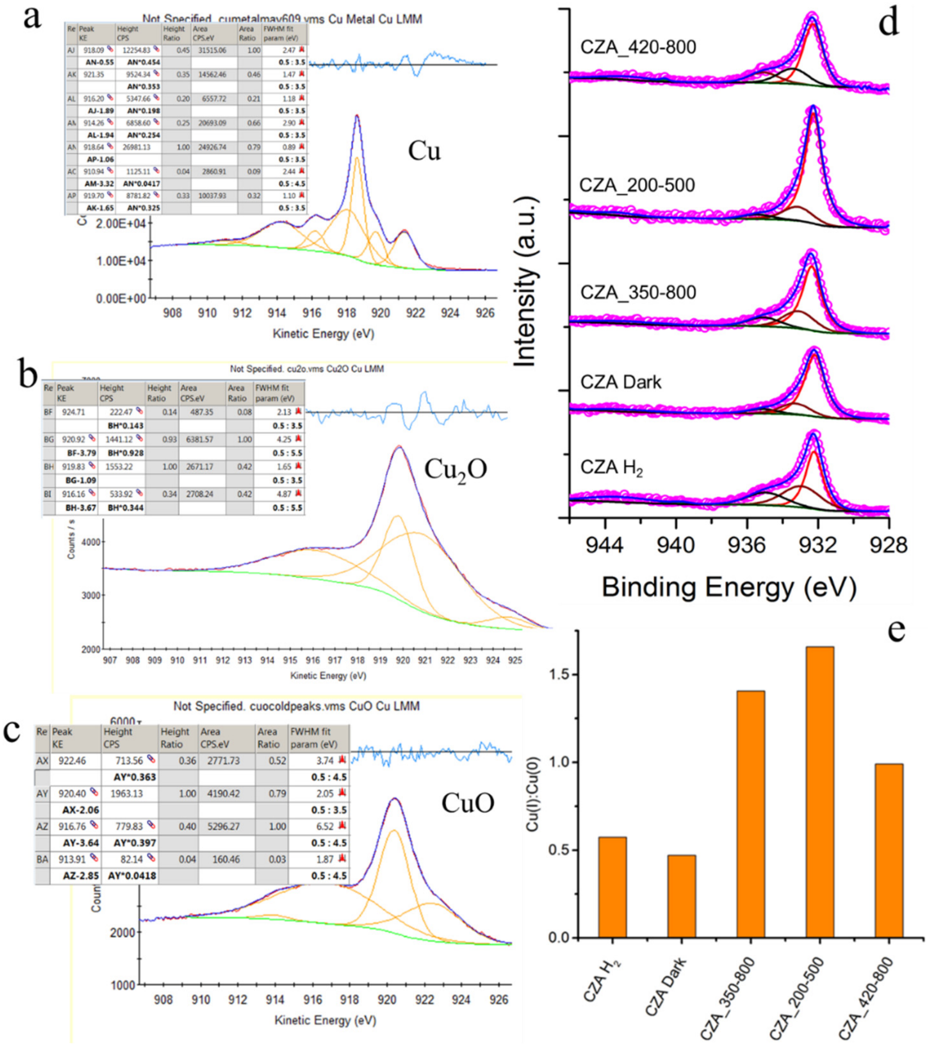

Supplementary Fig. 14. Method used for Cu species quantification. The curve-fitting results of reference spectra **(a-c),** XPS of Cu 2p (**d**), and calculated Cu(I):Cu(0) value (**e**). Insets provide details on parameters used for fitting. Deconvolution of the Cu LMM spectra was achieved using a linear fit combination based on the reference Auger spectra of Cu, Cu_2O, and CuO (from online open resources: http://www.xpsfitting.com/search/label/Copper) and peak fitting parameters reported by Biesinger[3]. Based on the peak separation information provided in Biesinger's study, we first conduct curve-fitting in reference Cu LMM spectra with fixed sub-peak positions relative to the one with highest kinetic energy (HKE) (take Cu_2O for example, HKE-3.79, HKE-4.88, HKE-7.58, and HKE were taken as the sub-peak positions, and position of HKE might change in different samples) and fixed range of FWHM parameter (initially 0.5-3.5, adjusted to 0.5-5.5 when necessary). Cu, Cu_2O, and CuO species were deconvoluted into seven, four, and four sub-peaks, respectively. Good fitting results were obtained with reference spectra as shown in Supplementary Figs. 14a-c. Afterwards, the peak height relations were recorded for further curve-fittings of sample spectra. (taken Cu_2O species again for example, the height for the above-mentioned four sub-peaks is I×0.143, I×0.928, I, I×0.344, respectively) Finally, the Cu speciation was accomplished with the deconvolution of Cu LMM spectra with fixed peak separation, relative peak height ratio, and FWHM range. Due to a large number of curves involved in the Cu LMM analysis, Cu 2p spectra (Supplementary Fig. 14d) were always taken as a reference to cross-check the [Cu(I)+Cu(0)]/Cu(II) ratio.

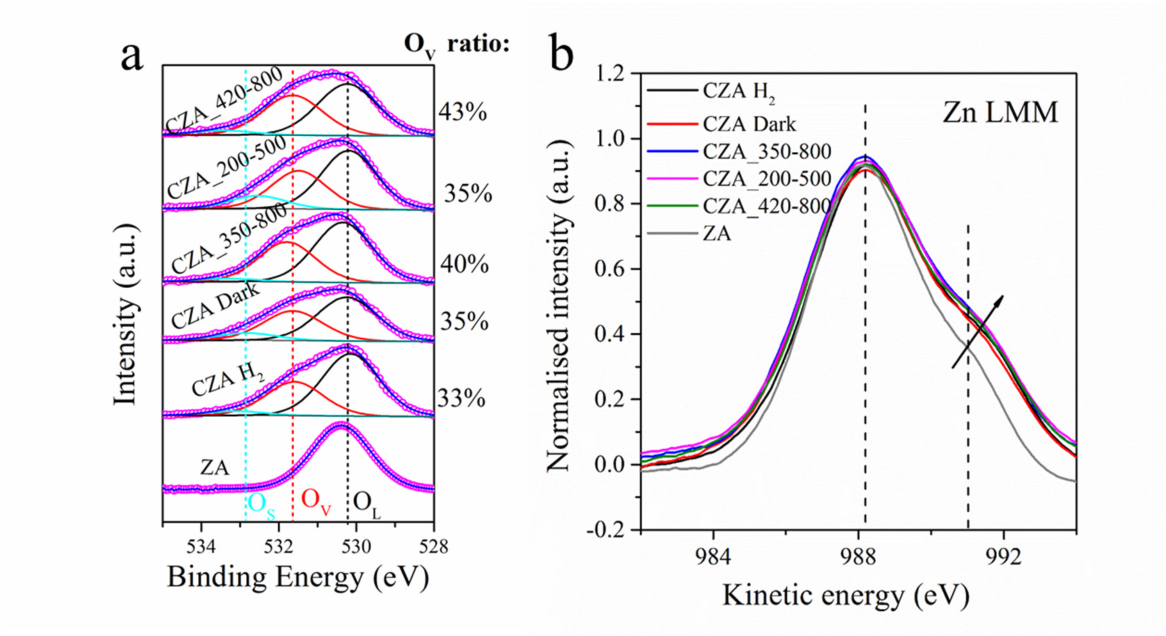

Supplementary Fig. 15. Surface defects on ZnO. XPS O1s (**a**) and Zn LMM (**b**) of samples. The surface oxygen (hydroxyl group, etc.), oxygen vacancy, and lattice oxygen were denoted as O_S, O_V, and O_L, respectively.

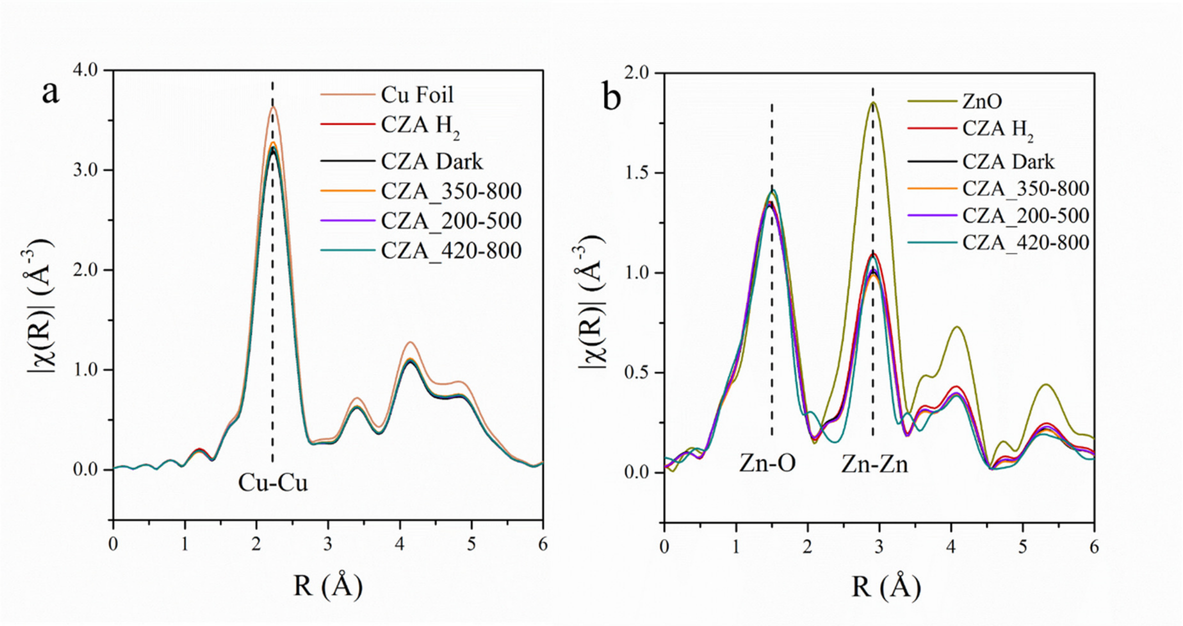

Supplementary Fig. 16. Fourier transform of EXAFS spectra. (**a**) Cu K edge and (**b**) Zn K edge for CZA spent catalysts.

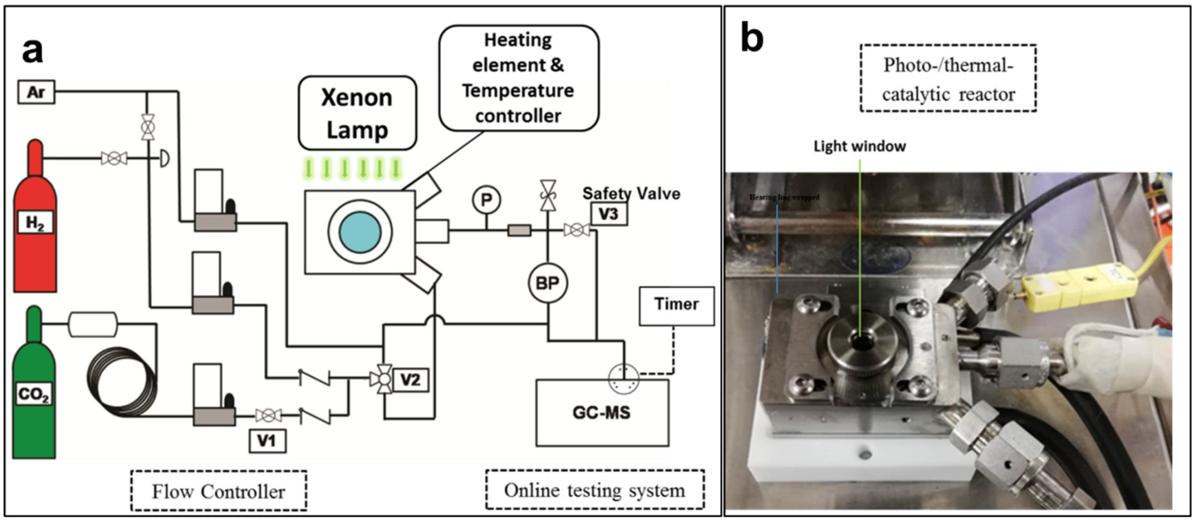

Supplementary Fig. 17. Configuration of flow photoreactor equipped with high pressure Harrick reactor (HVC-MRA-5, Harrick technology) with heating cartridge and K-type thermocouple. (**a**) Flow chart of the performance study system; (**b**) Picture of the reactor cell. Xenon lamp (which provided illumination) mounted on the top of a 13×2 mm SiO_2 window (distance of 6 cm between light source and window) - cold mirrors and/or a long pass filter were used to regulate the incident spectral range in the experiments.

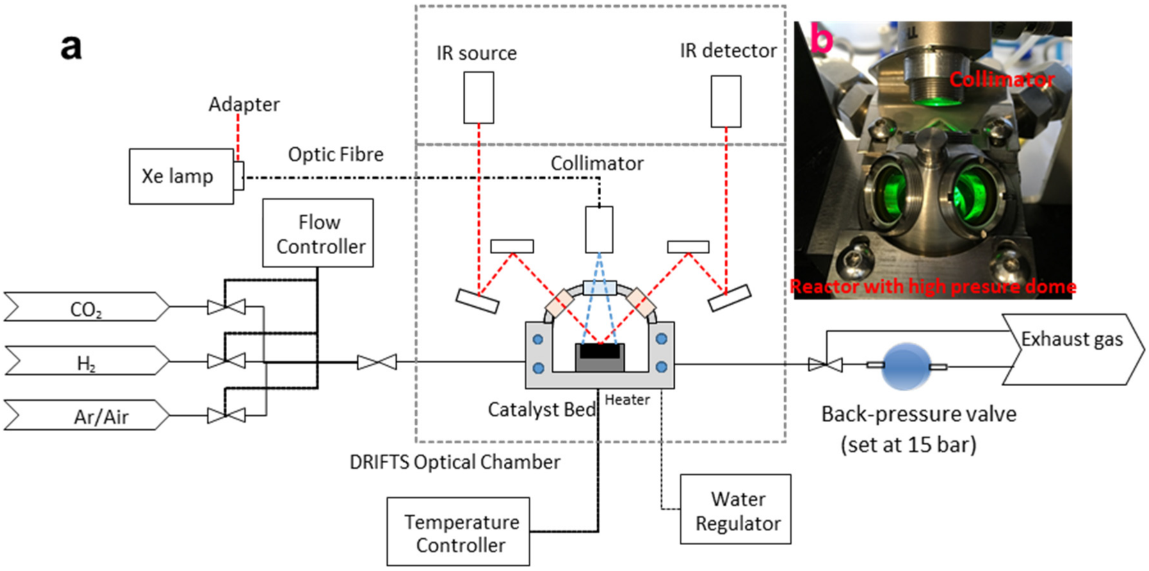

Supplementary Fig. 18. Schematic illustration of the *in-situ* high pressure (15 bar) DRIFTS analysis cell equipped with Xe lamp (350-800 nm) illumination. (**a**) Flow chart of the light-coupled high-pressure DRIFTS system; (**b**) Picture of the DRIFTS reactor cell.

Supplementary Table 1. Elemental ratio and Cu physical property. Cu/Zn/Al atomic ratio (determined from XPS), Cu dispersion (calculated from N_2O-chemisorption) and average Cu crystallite size (estimated from XRD) of CZA, CZ, CA and ZA.

Sample	Atomic ratio Cu/Zn/Al (%)	Cu dispersion (%)	Cu crystallite size (nm)
CZA	42.3/38.5/19.2	6.47	10.5
CZ	28.7/71.3/0	3.50	31.3
CA	44/0/56	1.64	17.6
ZA	0/67/33		

Supplementary Table 2. DRIFTS peak assignments.

Frequency (cm^{-1})	Assignment	Species	Reference
1593	ν_{as}(OCO)	ZnO-HCOO*	Sci. Adv. 2017[4], J. Catal. 2004[5], App. Catal., A 1992[6]
1375-1380, 1350	ν_{s}(OCO)		
1463-1492	carbonate	ZnO-CO_3*	J. Phys. Chem. B 1998[7]
1622	bicarbonate	ZnO-HCO_3*	Sci. Adv. 2017[4]
1795	formaldehyde	ZnO-H_2CO*	J. Phys. Chem. C 2018[8]
1050	$\nu(OCH_3)$	ZnO-H_3CO*	J. Phys. Chem. B 1998[7], Chem Phys Chem 2010[9]
1542-1550	ν_{as}(OCO)	Cu-HCOO*	J. Catal. 2004[5], Angew. Chem. Int. Ed. 2017[10]
1366	ν_{s}(OCO)		
1336, 1270	carbonate	Cu-CO_3*	Catal. Lett. 1994[11]
979	$\nu(OCH_3)$	Cu-H_3CO*	Catal. Lett. 1994[11]
2923, 2863	$\nu(CH_3)$		
1602	δ(OH)	Cu-H_2O	J. Catal. 2004[5], J. Catal. 1997[12]
2113	ν(CO)	Cu(I)-CO	J. Catal. 1997[12]
2077	ν(CO)	Cu-CO	J. Catal. 1997[12]
3017, 1306	$\nu(CH_4)$	gas CH_4	
2843, 1032	$\nu(CH_3OH)$	CH_3OH	Catal. Lett. 1994[11]

Supplementary Table 3. EXAFS modelling results from reference sample and catalysts treated under different conditions.

	Shell	N	$\Delta\sigma^2$ (10^{-3} Å^2)	R (Å)	ΔE_0 (eV)
Cu ref	Cu-Cu (1st)	12	8.6±0.7	2.54±0.01	0
	Cu-Cu (2nd)	6	14.6±4.1	3.59±0.01	
ZnO ref	Zn-O (1st)	4	5.1±2.3	1.98±0.01	0
	Zn-Zn/O (1st)	7	10.4±6.0	3.21±0.02	
	Zn-Zn (1st)	6	7.3±3.7	3.25±0.02	
	Zn-O (2nd)	24	15.6±6.0	3.81±0.03	
CZA H_2	Cu-Cu (1st)	10.7±0.4	8.8±0.3	2.54±0.00	0
	Cu-Cu (2nd)	30.9±9.1	32.9±4.5	3.59±0.00	
	Zn-O (1st)	3.7±0.4	4.6±1.6	1.98±0.01	0.006
	Zn-Zn/O (1st)	1.0±3.6	6.0±20.7	3.20±0.01	
	Zn-Zn (1st)	9.1±6.7	11.9±5.8	3.25±0.01	
	Zn-O (2nd)	9.6±4.5	8.9±8.6	3.80±0.01	
CZA dark	Cu-Cu (1st)	10.6±0.4	8.7±0.3	2.54±0.00	-0.062
	Cu-Cu (2nd)	30.9±8.7	33.1±4.3	3.59±0.00	
	Zn-O (1st)	3.6±0.4	4.6±1.8	1.98±0.01	0.309
	Zn-Zn/O (1st)	1.9±4.9	7.3±15.7	3.21±0.01	
	Zn-Zn (1st)	7.5±8.5	12.6±9.1	3.25±0.01	
	Zn-O (2nd)	9.5±5.0	9.3±10.2	3.81±0.01	
CZA full	Cu-Cu (1st)	11.1±0.4	8.8±0.3	2.54±0.00	0.059
	Cu-Cu (2nd)	32.5±8.4	33.3±3.9	3.59±0.00	
	Zn-O (1st)	3.7±0.4	4.6±1.6	1.98±0.01	-0.002
	Zn-Zn/O (1st)	1.7±4.4	7.5±15.5	3.21±0.01	
	Zn-Zn (1st)	7.4±7.4	12.4±7.8	3.25±0.01	
	Zn-O (2nd)	9.5±4.7	9.8±9.8	3.81±0.01	
CZA UV	Cu-Cu (1st)	10.8±0.4	8.7±0.3	2.54±0.00	-0.06
	Cu-Cu (2nd)	31.5±8.7	33.0±4.2	3.59±0.00	
	Zn-O (1st)	3.7±0.4	4.8±1.6	1.98±0.01	0.023
	Zn-Zn/O (1st)	1.4±4.0	6.9±16.1	3.21±0.01	
	Zn-Zn (1st)	7.9±6.8	12.2±6.7	3.25±0.01	
	Zn-O (2nd)	9.9±4.9	10.2±9.6	3.81±0.01	
CZA Vis	Cu-Cu (1st)	10.8±0.4	8.7±0.3	2.54±0.00	-0.051
	Cu-Cu (2nd)	31.6±8.6	33.1±4.1	3.59±0.00	
	Zn-O (1st)	3.7±0.5	5.0±1.8	1.98±0.01	0.335
	Zn-Zn/O (1st)	2.0±4.7	7.7±13.9	3.21±0.01	
	Zn-Zn (1st)	6.4±8.0	12.7±9.8	3.25±0.01	
	Zn-O (2nd)	9.9±5.7	11.1±11.5	3.81±0.01	

Supplementary Note 1

Additional characterisation results for catalysts:

As depicted in Supplementary Figs. 1a-e, Al_2O_3 (with ~10 at. % loading) is acting as a substrate for the growth of Cu/ZnO nanoparticles. Cu nanoparticles are surrounded by ZnO patches and the isolated unit is well-dispersed within Al_2O_3 support. After reaction (Supplementary Figs. 1f-j), ZnO nanoparticles were highly aggregated and interconnected with small-sized Cu species, which is supporting the dynamic feature of the particles and the migration of ZnO to the Cu surface under reaction conditions. In addition, the interactions among all species seem to be promoted since particle boundaries can hardly be distinguished and a porous cluster morphology, which may represent a stable structure of active catalyst was finally obtained. After introducing light into the system (Supplementary Fig. 3), there is a reconstruction (particle jointing) in the microstructure of the catalyst (denoted by red arrow in Supplementary Fig. 3h).

The presence of Cu metal and wurtzite ZnO were identified in the XRD patterns of the reduced sample (Supplementary Fig. 2a). Al_2O_3 exhibits amorphous-like structural features. Overlapping of the Cu_2O and ZnO peaks makes it difficult to determine the presence of Cu_2O in the XRD pattern. The small amount of preserved Cu oxide on the Cu particle surface is thought to be vital for the optimized chemical properties of the Cu-ZnO interface during CO_2 hydrogenation[13]. Compared to the Al-free catalyst (CZ), XRD pattern showed that alumina incorporation promotes an interaction between the different metal components, which is supported by the presence of weak aluminates[14] peaks in the XRD patterns of CA and CZA. The CO_2-TPD profiles (Supplementary Fig. 2b) show that, without ZnO, CA provides limited CO_2 adsorption sites, while the presence of defective ZnO favours CO_2 adsorption. A downward shift the in CO_2 desorption temperature by at least 150 °C is observed when comparing the profiles of ZA and CZA, where the desorption peak across the 400-450 °C region in ZA is shifted to 250 °C in CZA. The decrease in temperature is believed to arise from the destabilisation of CO_2 on the ZnO surface in the presence of Cu. This is essential for MeOH synthesis whereby the destabilisation of surface adsorbed CO_2 at low temperatures is critical for controlling the selectivity towards MeOH. Interaction between the Cu and ZnO is also apparent from the binding energy (BE) shifts in Cu 2p (negative-shift) and Zn 2p/O 1s (positive-shift) spectra (Supplementary Figs. 1c, d). This provides evidence of electron transfer from the ZnO to Cu in the CZA catalyst[15]. As shown in Fig. 1a-c and Supplementary Fig. 1, Al_2O_3 acts as a structural promoter for improving Cu dispersion (6.5% (CZA) *vs.* 3.5% (CZ)) as was determined by N_2O chemisorption experiments (Supplementary Table 1).The presence of aluminate improves the Cu dispersion, while ZnO could favourably promote CO_2 chemisorption and stimulate electronic interaction within the Cu component.

So as to provide information on the Cu size in different samples, we have included the HR-TEM image of CA. The size of Cu within the CA sample is around 10-20 nm. The averaged crystalline size of Cu was estimated to be around 17.6 nm from the XRD pattern in Supplementary Table 1 which agrees well with the HR-TEM image.

Supplementary Note 2

Selectivity *vs.* Temperature and activation energy for RWGS reaction:

Under the dark condition, the selectivity toward MeOH decreased due to promotion of the RWGS reaction as temperature increased. This can be correlated to the exothermic and endothermic natures of MeOH production and the RWGS reaction, respectively. Under illumination, CO production was enhanced across the considered temperature range (Supplementary Fig. 8c), while a significant increase in MeOH-production was only detected at 200-250 °C. As illustrated in Supplementary Fig. 11, this was attributed to the temperature-dependency of the formate conversion rate. Finally, a slightly altered although similar MeOH selectivity *vs.* temperature trend (when compared to the dark condition) was observed depending on the degree of light promotion in CO and MeOH production.

The calculated apparent activation energy for the RWGS reaction is shown in Supplementary Fig. 8d. A decrease in the apparent activation energy from 15.1 to 11.9 kcal/mol was observed under the irradiated condition. As revealed by Robatjazi et al.[16], generated electrons on accessible unpopulated adsorbate orbitals can activate the CO_2 reactant by either forming transient negatively charged ions or polarised species[16, 17], thus lowering the activation energy for the overall RWGS reaction[16, 18, 19]. In this study, a similar mechanism, where the electrons generated from Cu LSPR (or potentially transferred from excited ZnO) transiently occupy the orbital states of surface CO_2 to promote its activation and subsequent processes before decaying into thermal energy into the lattice, is used to explain the observed CO production performance under light irradiation (Supplementary Fig. 8c). In addition, CO production was found to be promoted across the considered temperature range under irradiation, which differs from MeOH production.

Supplementary Note 3

Contributions from photothermal heating and photocatalytic processes:

As elucidated in the main text, the photothermal CO_2 hydrogenation over $Cu/ZnO/Al_2O_3$ is essentially a light-assisted thermocatalysis where photochemical processes are synergistically contributing to the methanol synthesis. Absorbed photon energy (visible light) via the Cu LSPR can remain trapped inside the metal nanostructure and cause local heating of the Cu metal lattice surroundings, which may contribute to the CO_2 hydrogenation reaction. However, while the contribution from localised photothermal heating cannot be completely disregarded, to evaluate the extent of localised heating a control experiment where the reduced CZA sample was irradiated under the same reaction conditions (illumination without external heating) for two hours was conducted. There was a small temperature increase from 24 °C to 26 °C, indicating that heat generation within catalyst powder due to illumination is insignificant. Additionally, when comparing the MeOH yield and selectivity at 225 °C under both the dark (non-illuminated) and illuminated (350-800 nm light) conditions, while there is an enhanced MeOH yield the MeOH selectivity remains essentially unchanged. This implies that the enhanced MeOH yield under irradiation primarily derives from the photocatalytic process and not thermal effect. If the thermal effect was having a significant influence, any attributable increase in MeOH yield would be at the expense of MeOH selectivity which is not the case here.

The near-linear dependence of the MeOH production rate on light intensity is an indicator of a charge-carrier-driven[17, 20] reaction which differs from a thermally-driven reaction. The relationship suggests that the excited electrons can interact efficiently with surface adsorbates and a photocatalytic mechanism is responsible for the observed photo-enhancement. However, the distinctive roles of electrons from Cu LSPR-excitation and ZnO bandgap excitation in promoting the CO_2 hydrogenation reaction over $Cu/ZnO/Al_2O_3$, and the interplay between the two remain unknown.

Supplementary Note 4

Discussion on reaction mechanism over different catalysts:

The distinctive MeOH-production capacity reflected in the DRIFTS spectrum (predominantly at 1031 cm^{-1} in Supplementary Fig. 9) and the activity results (Supplementary Fig. 8) for CA, ZA (ZA show negligible methanol performance), and CZA can be accounted for by three different reaction pathways on three different catalysts:

(i) CA – a minor amount of carbonate, formate, and methoxy can form on the Cu surface. The limited CO_2 adsorption sites and ineffective methoxy formation on the pure Cu surface deliver a poor MeOH production capability compared to CZA;

(ii) ZA - MeOH preferably forms from the strongly reducing atmosphere (CO/H_2 mixture) and CO_2 is thought to be a catalyst poison as it can potentially oxidise the active sites (O vacancies in ZnO_{1-x}).[21] In addition, the hydrogenation of Zn-formate to methoxy has been reported as a very slow surface reaction[6]. This may account for the negligible MeOH production/methoxy formation on ZA;

(iii) CZA - the relatively better MeOH-production benefits from the presence of the Cu-ZnO interfacial interaction, facilitating the formation of formate and methoxy species. These two are widely deemed as stable active intermediates for MeOH generation by Cu-containing catalysts.[7, 11, 22, 23, 24]

In terms of CO production, the CO can form on both the ZnO and Cu surface (gaseous phase CO at 2113, 2176 cm^{-1} for the three samples). Two possible pathways are available for CO formation:

(i) CO forms mainly on the Cu surface from the dissociation of Cu-CO_3*, as a distinct peak is presented at 2077 cm^{-1} and 2094 cm^{-1} indicating adsorbed CO* species on the CA and CZA samples, respectively. The peak intensity of gaseous CO decreases in the following order: CA>CZA>ZA, indicating that Cu alone favours CO-production, as is consistent with a higher CO selectivity (lower MeOH selectivity) observed for the CA sample (Supplementary Fig. 8b);

(ii) The second pathway for CO production follows the decomposition of ZnO-HCOO*on the ZnO surface[6], supported by the presence of a weak peak at 2164 cm^{-1} (the adsorption of CO on ZnO is weak).

Supplementary Note 5

Supplementary Fig. 11 illustrates the following temperature-dependent characteristics of the CO_2 hydrogenation reaction:

i) At T <150 ºC, CO_2 is chemically adsorbed on ZnO in the form of bicarbonate or carbonate depending on temperature, which can then convert to formate, while its further hydrogenation is kinetically unfavourable;

ii) At the onset temperature of methanol-production (200 °C), formate species begin to slowly convert to carbonyl hydrides (formaldehyde, methoxy, etc.) and then to methanol. All the active species (including carbonate) are observable due to a very low overall reaction rate;

iii) At temperature in the range of 225-250 ºC, formate accumulates on the catalyst surface and its conversion represents the rate-limiting step in methanol synthesis. No carbonyl hydrides are observed in the DRIFTS spectra. CO_2 is chemically bonded to the ZnO surface in the form of carbonate due to its higher thermal stability compared to bicarbonate and hydroxyl groups. The attached formate species can be activated and hydrogenated with the aid of light-generated electrons;

iv) At temperatures greater than 250 ºC, the energy supplied from heating alone is sufficient to overcome the activation energy barrier of formate conversion. At such a high temperature, the thermal catalytic reaction is dominant. The observed temperature-dependence of the photocatalytic performance could be related to kinetic differences of formate conversion across the temperature range studied. Formate appeared as the dominant surface species over CZA at lower temperatures (Fig. 2b). However, the amount of formate is temperature-dependent and its conversion represents one of the kinetically-limiting steps at 200-250 ºC, which could be promoted through electronic transitions in the photo-excited adsorbate-nanoparticle systems[25, 26].

On the other hand, adsorbed CO was widely detected on the Cu-containing catalyst from 50-400 ºC (Supplementary Fig. 9) with photo-induced CO production clearly demonstrated across the full temperature range (Supplementary Fig. 8c), indicating a non-volcanic CO production behaviour, contrary to MeOH synthesis.

Supplementary Note 6
Valence band/DFT alignment:

The XPS valence band spectra provide the valence band electronic states of Cu and ZnO. The valence band electronic states between 4.0 eV to 8.0 eV mainly originate from the O 2p orbitals of ZnO[27], while the peak at ~10.5 eV is attributed to Zn 3d in Zn or ZnO[27]. Al_2O_3 valence band features were not indicated as they completely overlap with the ZnO valence band[28] and Al_2O_3 only contributes up to 10 % of the CZA catalyst with a minor contribution to the overall catalytic activity.

DFT calculations have been performed to determine the molecular orbital energy levels of various possible reaction intermediates. The molecular orbital energy levels are aligned against the Cu 3d DOS and the XPS VB spectra of CZA to identify the possible catalyst active sites that favour electron interaction between CZA catalyst surface and the surface adsorbed intermediate species.

In terms of chemical bond formation, while valence electrons are the ones involved, electronic interactions usually occur between valence band electrons (ground state) excited into the conduction band (excited state) of the catalysts and those in the higher energy molecular orbitals of the reactant or intermediate species. Chemical bond cleaving occurs when electrons are injected into the antibonding orbitals of the adsorbate molecules to achieve zero bond order. However, theoretical calculations to determine the energy levels of excited electrons for catalysts are non-trivial, especially when the catalysts are made up of multiple components of various structures and crystal phases. Clearly, this approach has its limitations, since only relatively rudimentary comparisons may be made by considering the gas phase adsorbate orbital levels unbound to the substrate. Given the structural complexity inherent in dealing with a multicomponent substrate, devising a suitable model substrate to explore the adsorption of reactants, products, and intermediates is not at all trivial and poses a major challenge for the computational modelling of such catalysts[29]. Hence, valence band analysis is used as an indicator to indirectly probe the catalyst behaviour and its electronic interactions with reactant and intermediate species.

Initial inspection of Fig. 3 reveals that for all of the reactants, products and intermediates, the lowest unoccupied molecular orbital (LUMO) levels all lie approximately within the range of the highest CZA valence bands below the Fermi level, implying that occupation of these orbitals is possible and thus implying a possible binding interaction with the substrate, in agreement with the observed surface species elucidated from the experimental studies. Closer inspection of the alignment of unoccupied levels of key adsorbates with substrate bands can further reveal insights into a potential mechanism of activation of reactant species.

From an electron and energy level perspective, electrons at the same energy level will be promoted by the same extent energetically upon excitation by the same amount of energy. Therefore, valence band electrons with a similar energy level as the critical molecular orbitals of reactant species can be promoted to the antibonding orbitals of the reactant species upon excitation, allowing electronic interactions between the catalyst and the reactant that lead to bond formation between the catalyst surface and the reactant (orbital overlapping), while weakening or breaking the native bonds of the reactant molecule.

By aligning the Fermi levels of the CZA catalyst valence band spectrum, Cu 3d orbitals PDOS, and the molecular orbitals of reactant species and intermediates, it is apparent that the majority of the species either interact with ZnO O 2p (~4-8 eV), Zn 3d (~10 eV), and Cu 3d (~2-4 eV), in particular the $d_{x^2-y^2}$ and d_{z^2} orbitals (~3 eV) for Cu 3d. The electronic interaction between ZnO/Cu and the CO_2 antibonding orbitals, as a result of exotic ZnO defects and photoexcitations, offers an explanation for the observed extent of CO_2 activation, and the subsequent increase in catalytic performance, i.e. ZA < CZA < CZA + mono-excitation < CZA + dual-excitation.

Supplementary Note 7

The deficiency in lattice oxygen and formation of chemisorbed carbon/oxygen groups on ZnO are predictable under CO_2/H_2 (1:3 gas flow ratio) reaction conditions. When compared with untreated ZnO/Al_2O_3 (ZA), the reduced and spent CZA catalysts displayed an increase in the intensity of the peak at around 991 eV in the Zn Auger spectra (Supplementary Fig. 15a). The intensity increase may originate from either oxygen vacancies or metallic Zn[30]. The presence of metallic Zn was reported by Behrens *et al.*[13, 31] to be unlikely during methanol-synthesis (at around 250 ºC) and is supported by the Zn 2p spectra (Fig. 4b) (ZnO (1021.7 eV) vs. Zn (1021.4 eV)). A richness in oxygen vacancy (O_V) and surface oxygen groups (O_S, hydroxyl groups and carbonates, for example)[32] is apparent in the reduced and spent CZA catalyst in comparison with the untreated ZA. A small increase in the oxygen vacancy ratio in spent catalyst illuminated catalyst could be used to explain the observed changes in adsorption feature A and B since the hybridization between Zn 4p/4sp and O1s is dependent on the neighboring coordination environment.

Supplementary Note 8

Electronic pathways under different irradiation conditions:

Judging from the energy band diagrams of Cu and ZnO, electron transfer from an excited Cu orbital state to the ZnO conduction band (refer to plasmon-mediated electron transfer (PMET)) and electron donation from ZnO to Cu (refer to photocatalyst-cocatalyst scheme) are both possible under Cu and ZnO excitation, respectively. These electrons could be channelled to contribute to the photocatalytic processes on Cu (H_2 cleavage and CO_2 dissociation) and the ZnO surface (activation of formate species). The variation in binding energy (BE) for Zn 2p (Fig. 4b) is taken as an indicator of the overall electron exchange in the ZnO lattice under the different reaction/illumination conditions. After reaction in the dark, a positive BE shift in the Zn 2p spectra was observed, and is attributed to an improved Cu-ZnO electronic interaction/interfacial contact (electron extraction from ZnO lattice to Cu occurs) during the CO_2 hydrogenation reaction. Upon ZnO excitation (200-500 nm), an enhanced electron transfer from ZnO to Cu is suggested by a tiny positive Zn BE shift (compared to the dark condition). Under visible light illumination (420-800 nm), the excited Cu state could inhibit this process. Under dual excitation of the Cu and ZnO, although it is hard to capture dynamic electron transfer information between the Cu and ZnO, a zero net change in the electron density of ZnO was indicated by the Zn 2p spectra (CZA_350-800 vs. CZA Dark). It is thought that electron transfer between Cu and ZnO was attenuated due to the presence of a repulsive force between two excited components. Nevertheless, surface reactions (H_2 cleavage/migration and HCOO* activation) for methanol production may be favourably accelerated in the presence of photogenerated electrons on the Cu and ZnO.

Supplementary References

1. Chiarello G, Colavita E, De Crescenzi M, Nannarone S. Reflection electron-energy-loss investigation of the electronic and structural properties of palladium. *Phys. Rev. B* **29**, 4878-4889 (1984).

2. Wang H, Tam F, Grady NK, Halas NJ. Cu Nanoshells: Effects of Interband Transitions on the Nanoparticle Plasmon Resonance. *J. Phys. Chem. B* **109**, 18218-18222 (2005).

3. Biesinger MC. Advanced analysis of copper X-ray photoelectron spectra. *Surface and Interface Analysis* **49**, 1325-1334 (2017).

4. Wang J*, et al.* A highly selective and stable ZnO-ZrO_2 solid solution catalyst for CO_2 hydrogenation to methanol. *Sci. Adv.* **3**, e1701290 (2017).

5. Yang R, Fu Y, Zhang Y, Tsubaki N. In situ DRIFT study of low-temperature methanol synthesis mechanism on Cu/ZnO catalysts from CO_2-containing syngas using ethanol promoter. *J. Catal.* **228**, 23-35 (2004).

6. Neophytides SG, Marchi AJ, Froment GF. Methanol synthesis by means of diffuse reflectance infrared Fourier transform and temperature-programmed reaction spectroscopy. *Appl. Catal., A* **86**, 45-64 (1992).

7. Chen H, Chen L, Lin J, Tan K, Li J. Comparative surface studies of high-Zn-level and commercial Cu/ZnO/Al_2O_3 catalysts. *J. Phys. Chem. B* **102**, 1994-2000 (1998).

8. Sápi A*, et al.* In Situ DRIFTS and NAP-XPS Exploration of the Complexity of CO_2 Hydrogenation over Size-Controlled Pt Nanoparticles Supported on Mesoporous NiO. *J. Phys. Chem. C* **122**, 5553-5565 (2018).

9. Kähler K, Holz MC, Rohe M, Strunk J, Muhler M. Probing the reactivity of ZnO and Au/ZnO nanoparticles by methanol adsorption: a TPD and DRIFTS study. *ChemPhysChem* **11**, 2521-2529 (2010).

10. Larmier K*, et al.* CO_2-to-Methanol Hydrogenation on Zirconia-Supported Copper Nanoparticles: Reaction Intermediates and the Role of the Metal–Support Interface. *Angew. Chem.* **129**, 2358-2363 (2017).

11. Bailey S, Froment GF, Snoeck JW, Waugh KC. A DRIFTS study of the morphology and surface adsorbate composition of an operating methanol synthesis catalyst. *Catal. Lett.* **30**, 99-111 (1994).

12. Fisher IA, Bell AT. *In-Situ* Infrared Study of Methanol Synthesis from H_2/CO_2 over Cu/SiO_2 and Cu/ZrO_2/SiO_2. *J. Catal.* **172**, 222-237 (1997).

13. Lunkenbein T*, et al.* Bridging the Time Gap: A Copper/Zinc Oxide/Aluminum Oxide Catalyst for Methanol Synthesis Studied under Industrially Relevant Conditions and Time Scales. *Angew. Chem.* **128**, 12900-12904 (2016).

14. Wang J, Zeng C. Al_2O_3 effect on the catalytic activity of Cu-ZnO-Al_2O_3-SiO_2 catalysts for dimethyl ether synthesis from CO_2 hydrogenation. *J. Nat. Gas Chem* **14**, 156-162 (2005).

15. Liao F*, et al.* Morphology-Dependent Interactions of ZnO with Cu Nanoparticles at the Materials' Interface in Selective Hydrogenation of CO_2 to CH_3OH. *Angew. Chem. Int. Ed.* **50**, 2162-2165 (2011).

16. Robatjazi H*, et al.* Plasmon-induced selective carbon dioxide conversion on earth-abundant aluminum-cuprous oxide antenna-reactor nanoparticles. *Nat. Commun.* **8**, 27 (2017).

17. Linic S, Aslam U, Boerigter C, Morabito M. Photochemical transformations on plasmonic metal nanoparticles. *Nat. Mater.* **14**, 567-576 (2015).

18. Upadhye AA*, et al.* Plasmon-enhanced reverse water gas shift reaction over oxide supported Au catalysts. *Catal. Sci. Technol.* **5**, 2590-2601 (2015).

19. Kim C*, et al.* Energy-efficient CO_2 hydrogenation with fast response using photoexcitation of CO_2 adsorbed on metal catalysts. *Nat. Commun.* **9**, 3027 (2018).

20. Christopher P, Xin H, Linic S. Visible-light-enhanced catalytic oxidation reactions on plasmonic silver nanostructures. *Nat. Chem.* **3**, 467 (2011).

21. Kurtz M*, et al.* Active Sites on Oxide Surfaces: ZnO-Catalyzed Synthesis of Methanol from CO and H_2. *Angew. Chem. Int. Ed.* **44**, 2790-2794 (2005).

22. Kobl K, Angelo L, Zimmermann Y, Sall S, Parkhomenko K, Roger A-C. In situ infrared study of formate reactivity on water–gas shift and methanol synthesis catalysts. *C. R. Chim.* **18**, 302-314 (2015).

23. Kattel S, Yan B, Yang Y, Chen JG, Liu P. Optimizing Binding Energies of Key Intermediates for CO_2 Hydrogenation to Methanol over Oxide-Supported Copper. *J. Am. Chem. Soc.* **138**, 12440-12450 (2016).

24. Nomura N, Tagawa T, Goto S. In situ FTIR study on hydrogenation of carbon dioxide over titania-supported copper catalysts. *Appl. Catal., A* **166**, 321-326 (1998).

25. Zhang H, Itoi T, Konishi T, Izumi Y. Dual Photocatalytic Roles of Light: Charge Separation at the Band Gap and Heat via Localized Surface Plasmon Resonance To Convert CO2 into CO over Silver–Zirconium Oxide. *J. Am. Chem. Soc.* **141**, 6292-6301 (2019).

26. Aslam U, Rao VG, Chavez S, Linic S. Catalytic conversion of solar to chemical energy on plasmonic metal nanostructures. *Nat. Catal.* **1**, 656 (2018).

27. Leontiev S, Koshcheev S, Devyatov V, Cherkashin A. Detailed XPS and UPS studies of the band structure of zinc oxide. *J. Struct. Chem.* **38**, 725 (1997).

28. Tao J, Chai J, Zhang Z, Pan J, Wang S. The energy-band alignment at molybdenum disulphide and high-k dielectrics interfaces. *Appl. Phys. Lett.* **104**, 232110 (2014).

29. Mora-Fonz D, Lazauskas T, Woodley SM, Bromley ST, Catlow CRA, Sokol AA. Development of interatomic potentials for supported nanoparticles: the Cu/ZnO case. *J. Phys. Chem. C* **121**, 16831-16844 (2017).

30. Schott V*, et al.* Chemische Aktivität von dünnen Oxidschichten: Starke Träger-Wechselwirkungen ergeben eine neue ZnO-Dünnfilmphase. *Angew. Chem.* **125**, 12143-12147 (2013).

31. Kandemir T*, et al.* In Situ Study of Catalytic Processes: Neutron Diffraction of a Methanol Synthesis Catalyst at Industrially Relevant Pressure. *Angew. Chem. Int. Ed.* **52**, 5166-5170 (2013).

32. Abliz A*, et al.* Rational design of ZnO: H/ZnO bilayer structure for high-performance thin-film transistors. *ACS Appl. Mater. Interfaces* **8**, 7862-7868 (2016).
