# Supplementary material for: Synergistic ultraviolet and visible light photo-activation enables intensified low-temperature methanol synthesis over copper/zinc oxide/alumina
Source: Nat Commun. 2020 Mar 31;11:1615. doi: 10.1038/s41467-020-15445-z (PMC7109065; doi:10.1038/s41467-020-15445-z)
Supplement: Supplementary file 2 — Peer Review [file 41467_2020_15445_MOESM2_ESM.pdf]

Reviewers' comments:

Reviewer #1 (Remarks to the Author):

The manuscript by Xie et al. reports light-enhanced CO_2 hydrogenation to methanol over the industrially-relevant $Cu/ZnO/Al_2O_3$ catalyst. The authors suggested that there is a synergy between Cu plasmonic excitation and ZnO band-gap excitation for the synthesis of methanol under UV-Vis light irradiation. Overall, this light-enhancement topic is of broad interest to the catalysis community. I also appreciate that many characterization techniques, such as XPS, XAS, DRIFTS and XANES, were used to investigate the effect of light on the CO_2 hydrogenation reaction. However, it seems to me some of the conclusions are not fully supported. This manuscript is potentially suitable for publication in Nat. Commun. after addressing the following issues:

1. The generality of the explanation is not very convincing without elucidating the temperature effect. Based on the dual photo-excitation mechanism proposed in this paper, why the light enhancement was only obvious at 225 ºC?
2. Photothermal effect of the Cu nanoparticles would lead to a higher local temperature than the measured temperature. This photothermal contribution seems overlooked in explaining the light effect.
3. As suggested by a latest review (Angew. Chem. Inter. Ed., DOI:10.1002/anie.201907443.), it is better to use "photo-thermo" instead of "photothermal" to avoid confusion. (Line 29, 103 and 356)
4. The effect of light intensity on the catalytic performance should be studied to help better understanding the photochemical and photothermal contributions.
5. The activation energy of the RWGS reaction under dark and light could provide very useful information. The effect of light the CO production was barely discussed in the manuscript.
6. In Line 266 to 268, "The positive BE shift was negated when CZA was illuminated with 420-800 nm light. This implies an electron transfer from Cu to ZnO under visible light irradiation." Please explain why this implies an electron transfer from Cu to ZnO, since the XPS peak of CZA_Dark appeared at a similar position with CZA_420-800.
7. When ZnO and Cu were excited at the same time, how do electrons flow from Cu to the conduction band of ZnO? There should be electrostatic repulsive force between excited electrons. More detailed analysis about this charge transfer is needed.
8. More information of CA, CZ, and ZA should be provided. For example, what is the size of Cu particles in CA?
9. There are also some typos list below:
(1) Line 122: An extra "at" was in the sentence.
(2) Line 209: I can't find "Figure 2d" in the main text.
(3) There are two "g" in Figure S2. Also, the scale bar in STEM image is not clear enough.

Reviewer #2 (Remarks to the Author):

The work is well done, accurate and uses advanced methods. It is timely and interedting. It is recomme ded to be published.

Reviewer #3 (Remarks to the Author):

The paper ” Synergistic UV and Visible Photo-activation Enables Intensified Low-temperature Methanol Synthesis over Cu/ZnO/Al2O3” is of high quality and presents new results that provides novel insights into the methanol synthesis reaction from carbon dioxide, and how this is lined to photo and thermal activation. I have one comment that, in my view, should be considered before publication.

The discussion would benefit from an extended analysis of the connection between selectivity and light irradiation. The selectivity seems to decrease throughout the entire temperature range tested, both in presence and absence of light.

I recommend publication after minor revision.

Manuscript ID: NCOMMS-19-37385A

Title: "Synergistic UV and Visible Photo-activation Enables Intensified Low-temperature Methanol Synthesis over Cu/ZnO/Al_2O_3"

Authors: *Bingqiao Xie[#], Roong Jien Wong[#], Tze Hao Tan, Michael Higham, Emma K. Gibson, Donato Decarolis, June Callison, Kondo-Francois Aguey-Zinsou, Michael Bowker, C. Richard A. Catlow, Jason Scott*, Rose Amal**

Response to Reviewer's Comments

Reviewer #1 (Remarks to the Author):

The manuscript by Xie et al. reports light-enhanced CO2 hydrogenation to methanol over the industrially-relevant Cu/ZnO/Al2O3 catalyst. The authors suggested that there is a synergy between Cu plasmonic excitation and ZnO band-gap excitation for the synthesis of methanol under UV-Vis light irradiation. Overall, this light-enhancement topic is of broad interest to the catalysis community. I also appreciate that many characterization techniques, such as XPS, XAS, DRIFTS and XANES, were used to investigate the effect of light on the CO2 hydrogenation reaction. However, it seems to me some of the conclusions are not fully supported. This manuscript is potentially suitable for publication in Nat. Commun. after addressing the following issues:

1. The generality of the explanation is not very convincing without elucidating the temperature effect. Based on the dual photo-excitation mechanism proposed in this paper, why the light enhancement was only obvious at 225 ºC?

Response: The limit in terms of word number meant that we could not include content relevant to the temperature effect within the main manuscript. Discussion relating to the effect of temperature on the extent of enhancement was presented on pages 16-17 of the original supporting information:

Figure S10 illustrates the following temperature-dependant characteristics of the CO_2 hydrogenation reaction:

i) At T < 150 ºC, CO_2 is chemically adsorbed on ZnO in the form of bicarbonate or carbonate depending on temperature, which can then convert to formate, while its further hydrogenation is kinetically unfavourable;

ii) At the onset temperature of methanol-production (200 °C), formate species begin to slowly convert to carbonyl hydrides (formaldehyde, methoxy, etc.) and then to methanol. All the active species (including carbonate) are observable due to a very low overall reaction rate;

iii) At temperature in the range of 225-250 ºC, formate accumulates on the catalyst surface and its conversion represents the rate-limiting step in methanol synthesis. No carbonyl hydrides are

observed in the DRIFTS spectra. CO_2 is chemically bonded to the ZnO surface in the form of carbonate due to its higher thermal stability compared to bicarbonate and hydroxyl groups. The attached formate species can be activated and hydrogenated with the aid of light-generated electrons;

iv) At temperatures greater than 250 ºC, the energy supplied from heating alone is sufficient to overcome the activation energy barrier of formate conversion. At such a high temperature, the thermal catalytic reaction is dominant. The observed temperature-dependence of the photocatalytic performance could be related to kinetic differences of formate conversion across the temperature range studied. Formate appeared as the dominant surface species over CZA at lower temperatures (Figure 2b). However, the amount of formate is temperature-dependent and its conversion represents one of the kinetically-limiting steps at 200-250 ºC, which could be promoted through electronic transitions in the photo-excited adsorbate-nanoparticle systems.

2. Photothermal effect of the Cu nanoparticles would lead to a higher local temperature than the measured temperature. This photothermal contribution seems overlooked in explaining the light effect.

Response: We have now included additional discussion on page 27 of the supporting information to explain the photothermal heating contribution:

As elucidated in the main text, the photothermal CO_2 hydrogenation over $Cu/ZnO/Al_2O_3$ is essentially a light-assisted thermocatalysis where photochemical processes are synergistically contributing to the methanol synthesis. Absorbed photon energy (visible light) via the Cu LSPR can remain trapped inside the metal nanostructure and cause local heating of the Cu metal lattice surroundings, which may contribute to the CO_2 hydrogenation reaction. However, while the contribution from localised photothermal heating cannot be completely disregarded, to evaluate the extent of localised heating a control experiment where the reduced CZA sample was irradiated under the same reaction conditions (illumination without external heating) for two hours was conducted. There was a small temperature increase from 24 °C to 26 °C, indicating that heat generation within catalyst powder due to illumination is insignificant. Additionally, when comparing the MeOH yield and selectivity at 225 °C under both the dark (non-illuminated) and illuminated (350-800 nm light) conditions, while there is an enhanced MeOH yield the MeOH selectivity remains essentially unchanged. This implies that the enhanced MeOH yield under irradiation primarily derives from the photocatalytic process and not thermal effect. If the thermal effect was having a significant influence, any attributable increase in MeOH yield would be at the expense of MeOH selectivity which is not the case here.

3. As suggested by a latest review (Angew. Chem. Inter. Ed., DOI:10.1002/anie.201907443.), it is better to use "photo-thermo" instead of "photothermal" to avoid confusion. (Line 29, 103 and 356)

Response: As mentioned by the reviewer, the term "photo-thermo catalysis" was used in review paper Angew. Chem. Inter. Ed., DOI:10.1002/anie.201907443 to describe the case where photocatalysis and thermocatalysis synergistically contribute to the catalytic reaction. Adding to the confusion, the term "plasmonic photo-thermocatalysis" has been used in another review paper – Adv. Mater., DOI:10.1002/adma.201600305 – to describe the photo-assisted catalytic process that is dominated solely by the photothermal effect of plasmonic nanometals, without an additional photoactivation mechanism resulting from hot-electron transfer and injection. Thus, using "photo-thermo" can also invoke confusion as to the phenomenon it is referring to. Consequently, in our case, we have retained "photothermal catalysis" to refer to the light-assisted thermocatalysis where photochemical processes are synergistically contributing to the methanol synthesis, with this definition now being included on page 27 of the revised supporting information.

In support of our retention of "photothermal catalysis" the same term was used in a recent review paper by Geoffrey Ozin and co-workers (Energy Environ. Sci., DOI:10.1039/C8EE02790K) to describe the catalytic process which involves the integration of both photochemical and thermochemical processes.

4. The effect of light intensity on the catalytic performance should be studied to help better understanding the photochemical and photothermal contributions.

Response: As suggested by Reviewer 1, the reaction rate of MeOH under varied light intensity was evaluated and is now presented as Figure S16 (below) in the Supporting Information (page 27).

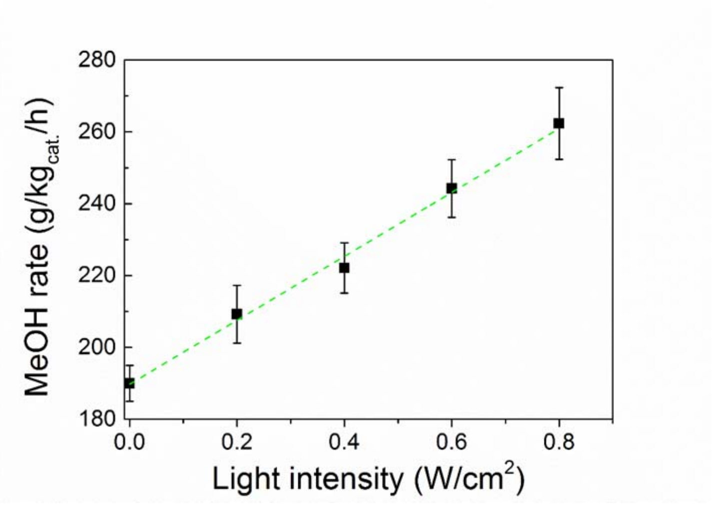

Figure S16. Intensity-dependent photothermal MeOH production rate. The dashed line shows a near-linear relation between rate and light intensity (R^2=0.994).

The near-linear dependence of the MeOH production rate on light intensity is an indicator of a charge-carrier-driven[1-2] reaction which differs from a thermally-driven reaction. The relationship suggests that the excited electrons can interact efficiently with surface adsorbates and a photocatalytic mechanism is responsible for the observed photo-enhancement. However, the distinctive roles of electrons from Cu LSPR-excitation and ZnO bandgap excitation in promoting the CO_2 hydrogenation reaction over Cu/ZnO/Al_2O_3, and the interplay between the two remain unknown.

5. The activation energy of the RWGS reaction under dark and light could provide very useful information. The effect of light the CO production was barely discussed in the manuscript.

Response: The discussion on CO production (under dark and irradiated conditions) was presented in the Supporting Information (page 15):

In terms of CO production, the CO can form on both the ZnO and Cu surface (gaseous phase CO at 2113, 2176 cm^{-1} for the three samples). Two possible pathways are available for CO formation:

(i) CO forms mainly on the Cu surface from the dissociation of Cu-CO_3*, as a distinct peak is presented at 2077 cm^{-1} and 2094 cm^{-1} indicating adsorbed CO* species on the CA and CZA samples, respectively. The peak intensity of gaseous CO decreases in the following order: CA>CZA>ZA, indicating that Cu alone favours CO-production, as is consistent with a higher CO selectivity (lower MeOH selectivity) observed for the CA sample (Figure S7b);

(ii) The second pathway for CO production follows the decomposition of ZnO-HCOO*on the ZnO surface[3], supported by the presence of a weak peak at 2164 cm^{-1} (the adsorption of CO on ZnO is weak).

As demonstrated by the photothermal catalytic performance and surface chemistry results in the main text, CO production was photo-enhanced on the Cu surface by photo-generated electrons from either Cu LSPR or electrons transferring from ZnO due to bandgap excitation. Consequently, this led to an increase in the Cu-O ratio for the illuminated samples.

We have now calculated the apparent activation energy (E_a) for the RWGS reaction (Figure S7d) and included additional discussion to elucidate the effect of light on CO production (see Page 12 of the supporting information):

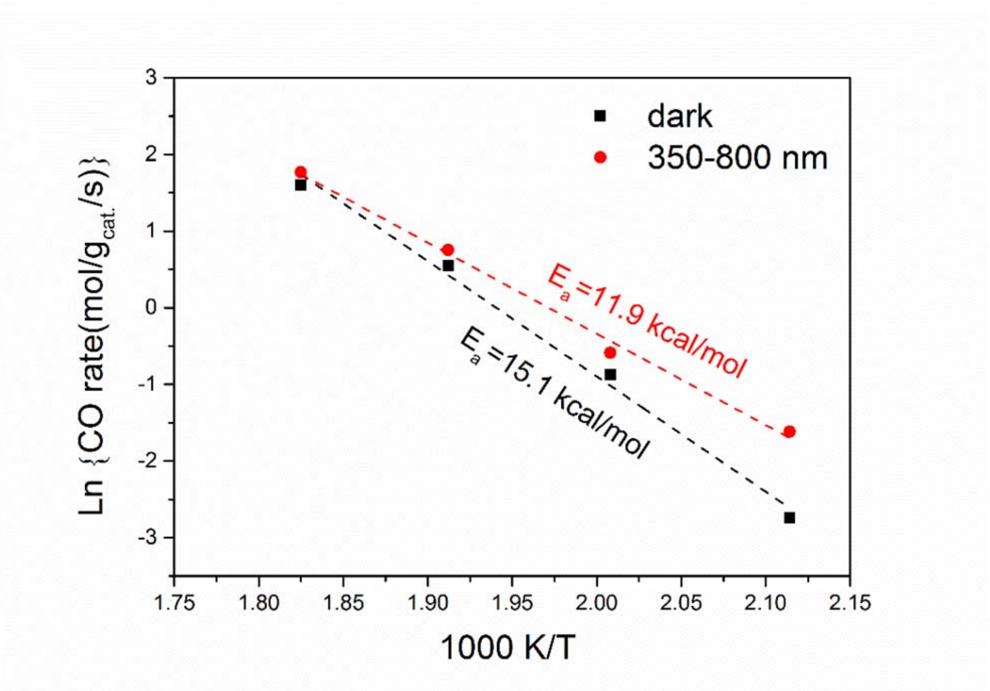

Figure S7d. Calculated activation energy for the RWGS reaction under dark and light conditions.

The calculated apparent activation energy for the RWGS reaction is shown in Figure S7d. A decrease in the apparent activation energy from 15.1 to 11.9 kcal/mol was observed under the irradiated condition. As revealed by Robatjazi et al.[4], generated electrons on accessible unpopulated adsorbate orbitals can activate the CO_2 reactant by either forming transient negatively charged ions or polarised species[2, 4], thus lowering the activation energy for the overall RWGS reaction[4-6]. In this study, a similar mechanism, where the electrons generated from Cu LSPR (or potentially transferred from excited ZnO) transiently occupy the orbital states of surface CO_2 to promote its activation and subsequent processes before decaying into thermal energy into the lattice, is used to explain the observed CO production performance under light irradiation (Figure S7c). In addition, CO production was found to be promoted across the considered temperature range under irradiation (Figure S7c), which differs from MeOH production.

6. In Line 266 to 268, “The positive BE shift was negated when CZA was illuminated with 420-800 nm light. This implies an electron transfer from Cu to ZnO under visible light irradiation.” Please explain why this implies an electron transfer from Cu to ZnO, since the XPS peak of CZA_Dark appeared at a similar position with CZA_420-800.

Response: Previously, the peak maximum was hard to determine since we did not conduct the peak-fitting analysis. After doing so, it becomes clearer that the Zn 2p binding energy (BE) for CZA_420-800 is not at a similar position to that for CZA_Dark. The relevant information has now been revised and is highlighted in the revised manuscript (beneath figure 4 as per below):

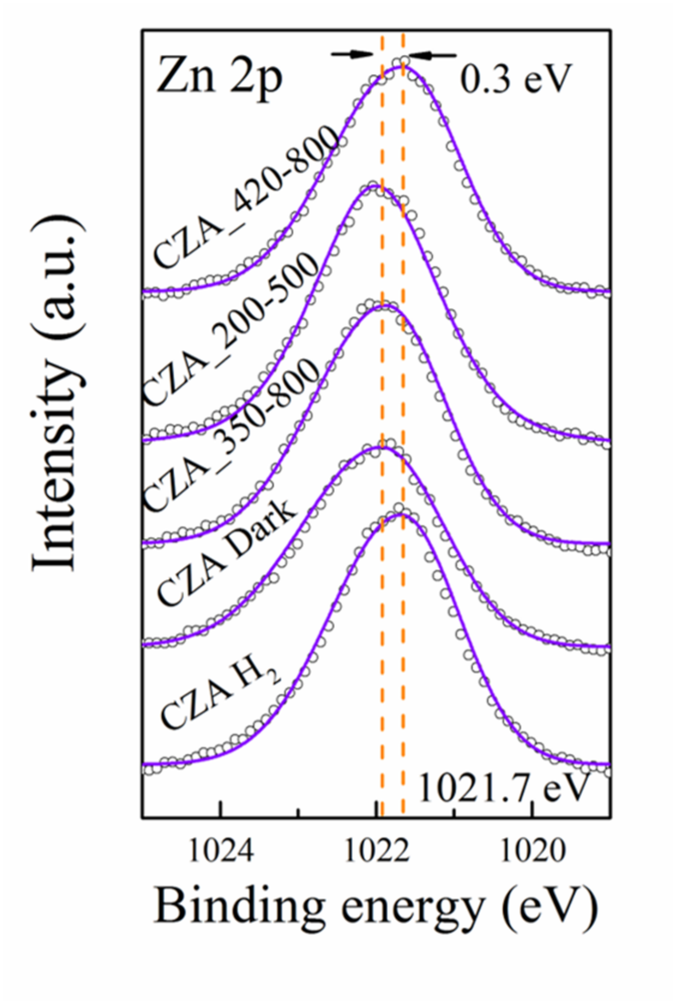

Figure 4b. Zn 2p spectra for reduced CZA catalyst and spent catalysts under different irradiation conditions.

The variation in binding energy (BE) for Zn 2p (Figure 4b) is taken as an indicator of the overall electron exchange in the ZnO lattice under different reaction conditions. Upon careful inspection, it

can be seen that the Zn 2p BE for CZA_420-800 is similar to that of the reduced catalyst (CZA H_2), but lower (by ~0.3 eV) than that of the spent catalysts under non-illuminated conditions, under irradiation by 200-500 nm light, and under irradiation by 350-800 nm light. The findings indicate that for the latter three reaction conditions, a net electron transfer from ZnO to Cu could occur, while under visible light illumination (420-800 nm), the excited Cu state could inhibit this process.

Please also refer to the Response to question 7 for further details.

7. When ZnO and Cu were excited at the same time, how do electrons flow from Cu to the conduction band of ZnO? There should be electrostatic repulsive force between excited electrons. More detailed analysis about this charge transfer is needed.

Response: It is not our intention to suggest that electrons flow from Cu to the conduction band of ZnO under visible light illumination. Upon irradiation by 350-800 nm light, no significant Zn 2p BE shift in CZA_350-800 relative to CZA Dark was observed, indicating there is no significant net electron exchange between Cu and ZnO under this condition. However, the generated electrons may participate in surface reactions. A more detailed discussion is provided below which has now been included in the revised supporting information section (page 28):

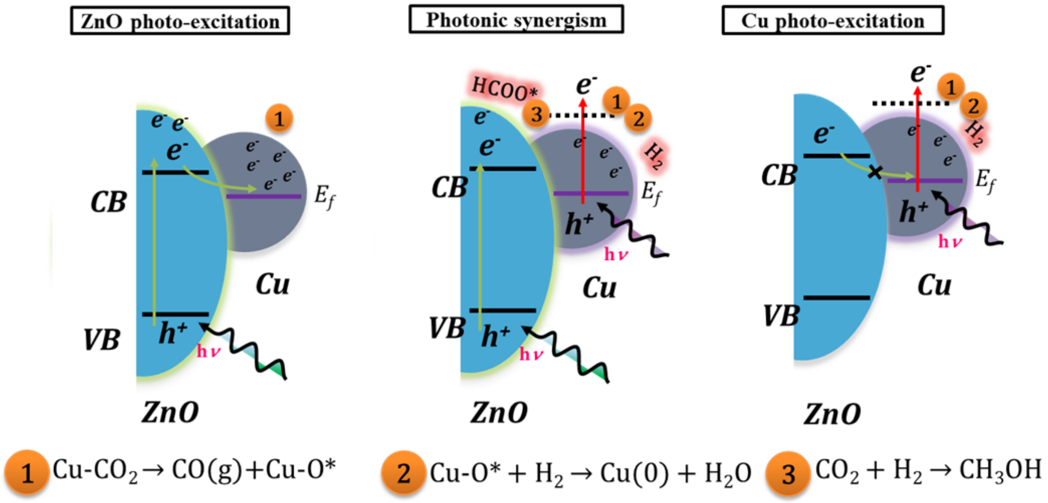

Figure 6a. Proposed mechanism illustrating electron transfer between Cu and ZnO under irradiation by different light spectral ranges; the reactions below the schematics indicate the potential reactions which could be improved when interacting with certain light-generated electrons.

Judging from the energy band diagrams of Cu and ZnO, electron transfer from an excited Cu orbital state to the ZnO conduction band (refer to plasmon-mediated electron transfer (PMET)) and electron donation from ZnO to Cu (refer to photocatalyst-cocatalyst scheme) are both possible under Cu and ZnO excitation, respectively. These electrons could be channelled to contribute to the photocatalytic

processes on Cu (H_2 cleavage and CO_2 dissociation) and the ZnO surface (activation of formate species). The variation in binding energy (BE) for Zn 2p (Figure 4b) is taken as an indicator of the overall electron exchange in the ZnO lattice under the different reaction/illumination conditions. After reaction in the dark, a positive BE shift in the Zn 2p spectra was observed, and is attributed to an improved Cu-ZnO electronic interaction/interfacial contact (electron extraction from ZnO lattice to Cu occurs) during the CO_2 hydrogenation reaction. Upon ZnO excitation (200-500 nm), an enhanced electron transfer from ZnO to Cu is suggested by a tiny positive Zn BE shift (compared to the dark condition). Under visible light illumination (420-800 nm), the excited Cu state could inhibit this process. Under dual excitation of the Cu and ZnO, although it is hard to capture dynamic electron transfer information between the Cu and ZnO, a zero net change in the electron density of ZnO was indicated by the Zn 2p spectra (CZA_350-800 vs. CZA Dark). It is thought that electron transfer between Cu and ZnO was attenuated due to the presence of a repulsive force between two excited components. Nevertheless, surface reactions (H_2 cleavage/migration and HCOO* activation) for methanol production may be favourably accelerated in the presence of photogenerated electrons on the Cu and ZnO.

8. More information of CA, CZ, and ZA should be provided. For example, what is the size of Cu particles in CA?

Response: We have previously provided information of CA, CZ, and ZA on Page 4 of original supporting information:

Compared to the Al-free catalyst (CZ), XRD pattern showed that alumina incorporation promotes an interaction between the different metal components, which is supported by the presence of weak aluminate peaks in the XRD patterns of CA and CZA. The CO_2-TPD profiles (Figure S1b) show that, without ZnO, CA provides limited CO_2 adsorption sites, while the presence of defective ZnO favours CO_2 adsorption. A downward shift the in CO_2 desorption temperature by at least 150 °C is observed when comparing the profiles of ZA and CZA, where the desorption peak across the 400-450 °C region in ZA is shifted to 250 °C in CZA. The decrease in temperature is believed to arise from the destabilisation of CO_2 on the ZnO surface in the presence of Cu. This is essential for MeOH synthesis whereby the destabilisation of surface adsorbed CO_2 at low temperatures is critical for controlling the selectivity towards MeOH. Interaction between the Cu and ZnO is also apparent from the binding energy (BE) shifts in Cu 2p (negative-shift) and Zn 2p/O 1s (positive-shift) spectra (Figure S1c and S1d). This provides evidence of electron transfer from the ZnO to Cu in the CZA catalyst. As shown in Figure 1a-c and S2, Al_2O_3 acts as a structural promoter for improving Cu dispersion (6.5% (CZA) *vs.* 3.5% (CZ)) as was determined by N_2O chemisorption experiments (Table S1).

So as to provide information on the Cu size in different samples, we have now included the below HR-TEM image of CA. The size of Cu within the CA sample is around 10-20 nm. The averaged crystalline size of Cu was estimated to be around 17.6 nm from the XRD pattern in Table S1 which agrees well with the HR-TEM image.

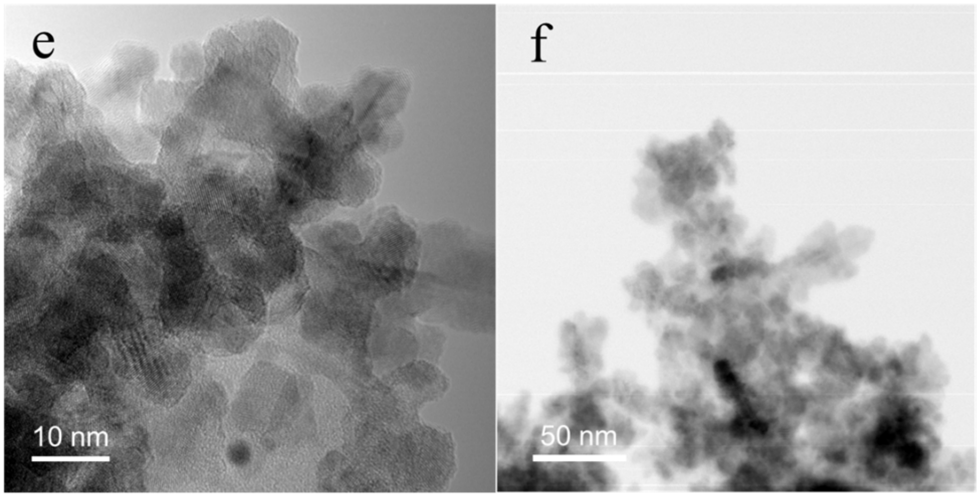

Figure S1. HR-TEM images of the reduced CA sample.

9. There are also some typos list below:

(1) Line 122: An extra “at” was in the sentence.

(2) Line 209: I can’t find “Figure 2d” in the main text.

(3) There are two “g” in Figure S2. Also, the scale bar in STEM image is not clear enough.

Response: We thank the reviewer for identifying the errors. They have now been corrected within the manuscript and Supporting Information.

Reviewer #2 (Remarks to the Author):

The work is well done, accurate and uses advanced methods. It is timely and interesting. It is recommended to be published.

Response: We thank the reviewer for their positive comment.

Reviewer #3 (Remarks to the Author):

The paper ” Synergistic UV and Visible Photo-activation Enables Intensified Low-temperature Methanol Synthesis over Cu/ZnO/Al2O3” is of high quality and presents new results that provides novel insights into the methanol synthesis reaction from carbon dioxide, and how this is lined to photo and thermal activation. I have one comment that, in my view, should be considered before publication.

The discussion would benefit from an extended analysis of the connection between selectivity and light irradiation. The selectivity seems to decrease throughout the entire temperature range tested, both in presence and absence of light.

Response: We thank the reviewer for their positive comments and constructive suggestions. We have now added additional discussion in the supplementary information section (page 11) based on the comment by Reviewer 3:

Under the dark condition, the selectivity toward MeOH decreased due to promotion of the RWGS reaction as temperature increased. This can be correlated to the exothermic and endothermic natures of MeOH production and the RWGS reaction, respectively. Under illumination, CO production was enhanced across the considered temperature range (Figure S7c), while a significant increase in MeOH-production was only detected at 200-250 °C. As illustrated in Figure S10 (and associated discussion), this was attributed to the temperature-dependency of the formate conversion rate. Finally, a slightly altered although similar MeOH selectivity *vs.* temperature trend (when compared to the dark condition) was observed depending on the degree of light promotion in CO and MeOH production.

Related references:

1. Christopher, P.; Xin, H.; Linic, S., Visible-light-enhanced catalytic oxidation reactions on plasmonic silver nanostructures. *Nature Chemistry* **2011,** *3*, 467.
2. Linic, S.; Aslam, U.; Boerigter, C.; Morabito, M., Photochemical transformations on plasmonic metal nanoparticles. *Nature Materials* **2015,** *14* (6), 567-576.

3. Neophytides, S. G.; Marchi, A. J.; Froment, G. F., Methanol synthesis by means of diffuse reflectance infrared Fourier transform and temperature-programmed reaction spectroscopy. *Applied Catalysis A: General* **1992,** *86* (1), 45-64.
4. Robatjazi, H.; Zhao, H.; Swearer, D. F.; Hogan, N. J.; Zhou, L.; Alabastri, A.; McClain, M. J.; Nordlander, P.; Halas, N. J., Plasmon-induced selective carbon dioxide conversion on earth-abundant aluminum-cuprous oxide antenna-reactor nanoparticles. *Nature Communications* **2017,** *8* (1), 27.
5. Upadhye, A. A.; Ro, I.; Zeng, X.; Kim, H. J.; Tejedor, I.; Anderson, M. A.; Dumesic, J. A.; Huber, G. W., Plasmon-enhanced reverse water gas shift reaction over oxide supported Au catalysts. *Catalysis Science & Technology* **2015,** *5* (5), 2590-2601.
6. Kim, C.; Hyeon, S.; Lee, J.; Kim, W. D.; Lee, D. C.; Kim, J.; Lee, H., Energy-efficient CO 2 hydrogenation with fast response using photoexcitation of CO 2 adsorbed on metal catalysts. *Nature Communications* **2018,** *9* (1), 3027.

REVIEWERS' COMMENTS:

Reviewer #1 (Remarks to the Author):

The authors have improved the quality of the manuscript after revision. Therefore, I would like to suggest the publication of the current version.

Response to Reviewer's Comments

Reviewer #1 (Remarks to the Author):

The authors have improved the quality of the manuscript after revision. Therefore, I would like to suggest the publication of the current version.

Response: We appreciate the positive feedback from reviewer.
